# Hitting a Moving Target: A Model for Malaria Elimination in the Presence of Population Movement

**DOI:** 10.1371/journal.pone.0144990

**Published:** 2015-12-21

**Authors:** Sheetal Prakash Silal, Francesca Little, Karen Irma Barnes, Lisa Jane White

**Affiliations:** 1 Department of Statistical Sciences, University of Cape Town, Rondebosch, 7700, South Africa; 2 Division of Clinical Pharmacology, Department of Medicine, University of Cape Town, Observatory, 7925, South Africa; 3 Mahidol Oxford Tropical Medicine Research Unit, Mahidol University, Bangkok, Thailand; 4 Centre for Tropical Medicine, Nuffield Department of Clinical Medicine, Churchill Hospital, University of Oxford, Oxford, United Kingdom; Swiss Tropical and Public Health Institute, SWITZERLAND

## Abstract

South Africa is committed to eliminating malaria with a goal of zero local transmission by 2018. Malaria elimination strategies may be unsuccessful if they focus only on vector biology, and ignore the mobility patterns of humans, particularly where the majority of infections are imported. In the first study in Mpumalanga Province in South Africa designed for this purpose, a metapopulation model is developed to assess the impact of their proposed elimination-focused policy interventions. A stochastic, non-linear, ordinary-differential equation model is fitted to malaria data from Mpumalanga and neighbouring Maputo Province in Mozambique. Further scaling-up of vector control is predicted to lead to a minimal reduction in local infections, while mass drug administration and focal screening and treatment at the Mpumalanga-Maputo border are predicted to have only a short-lived impact. Source reduction in Maputo Province is predicted to generate large reductions in local infections through stemming imported infections. The mathematical model predicts malaria elimination to be possible only when imported infections are treated before entry or eliminated at the source suggesting that a regionally focused strategy appears needed, for achieving malaria elimination in Mpumalanga and South Africa.

## Introduction

Mathematical modelling is an integral tool aiding our understanding of the dynamics of infectious diseases [1]. Mathematical models, and their applications to malaria in particular, have a history that spans over 100 years [2]. Since the call in October 2007 for renewed efforts towards achieving global malaria eradication, more than 25 previously endemic countries are in the pre-elimination or elimination phase of the eradication effort [3, 4]. As South Africa—now in the pre-elimination phase (<5 cases per 1000 people)—is committed to eliminating malaria by 2018, efforts are increasing in the malaria-endemic provinces, including Mpumalanga, beyond those needed for malaria control [5]. With vector-borne diseases like malaria, strategies to eliminate may be unsuccessful if they focus only on the vector and parasite biology and ignore the mobility patterns of humans [6]. This is particularly true in areas where the majority of infections are imported. Here, the elimination strategy needs to consider sources of infection in neighbouring regions, including mobility between regions and whether their control or elimination efforts are optimal. In this paper, a metapopulation, non-linear, stochastic, ordinary-differential equation model is used to simulate malaria transmission in Mpumalanga and neighbouring Maputo Province in Mozambique, in order to assess the potential impact of implementing the policy changes that may be used to achieve malaria elimination in Mpumalanga.

**Fig 1:**
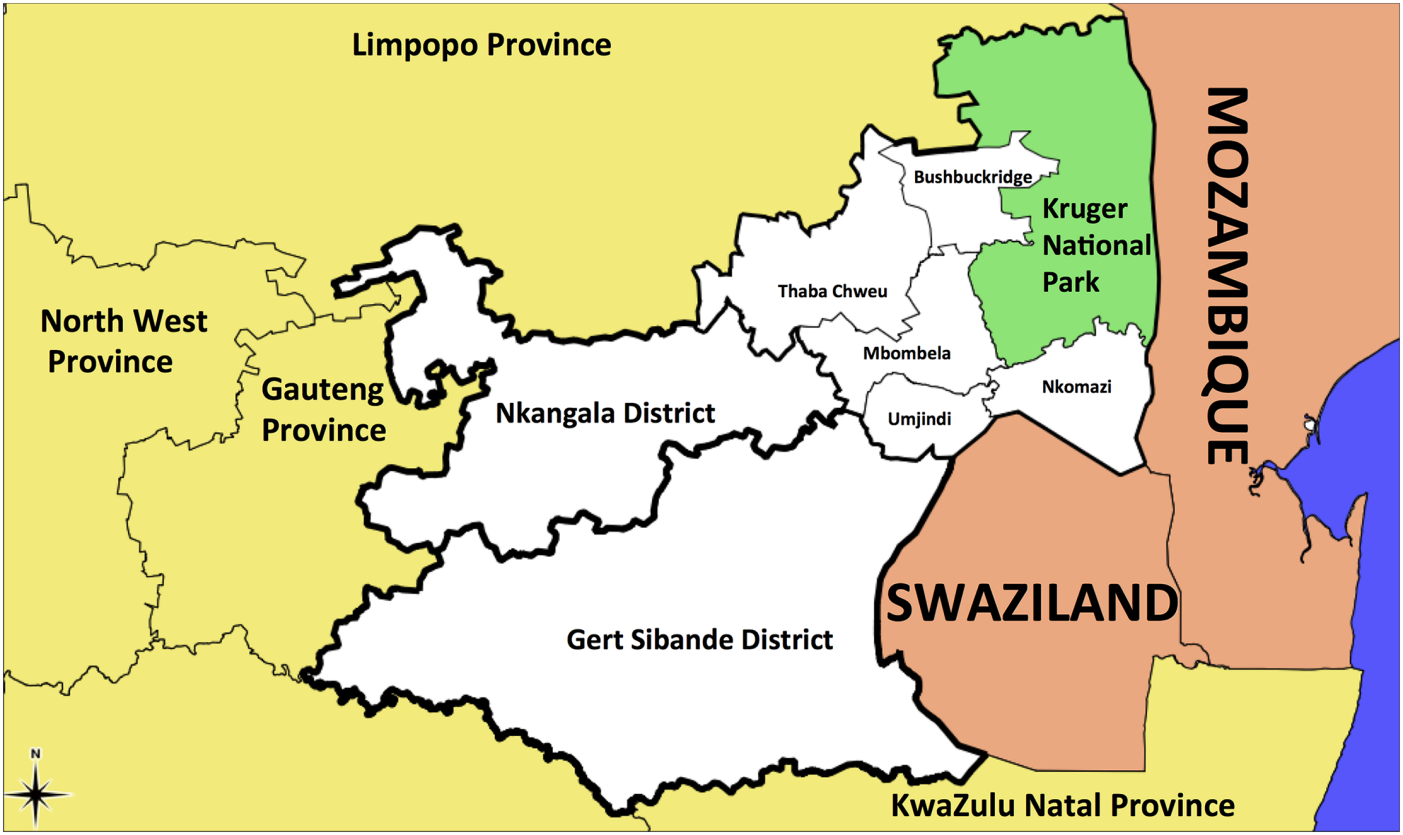
A map of Mpumalanga Province in relation to Mozambique and Swaziland (Source: Mpumalanga Malaria Elimination Programme (unpublished)).

Malaria prevalence and control in Mpumalanga has been documented extensively [7–13]. The five municipalities in the Ehlanzeni District bordering Maputo Province and Swaziland are those most affected by malaria in the province (Fig 1). Indoor residual spraying (IRS), the 2003 introduction of artemisinin-based combination therapy (ACT) of artesunate with sulphadoxine-pyremethamine, followed by artemether-lumefantrine (AL) in 2006, and the Lubombo Spatial Development cross-border Initiative (LSDI) are all considered responsible for the substantial decrease in malaria cases and malaria deaths in Mpumalanga since 2000 [7]. Between 2002 and 2012, 40 650 cases were notified, with the proportion of cases imported increasing from 39% in 2002 to 87% in 2012. Of the cases imported in 2012, 13% were sourced in South Africa, 85% from Mozambique and the remaining 2% from other African and Asian countries. Malaria is considered the most important public health problem in Mozambique accounting for 29% of all deaths, followed closely by AIDS at 27% [14].

Maputo Province, which shares the eastern border of Mpumalanga, has also experienced a sharp decline in malaria cases since 2002 but still has substantially higher malaria incidence. The LSDI malaria control programme was a regional collaboration between South Africa, Swaziland and Mozambique that aimed to decrease malaria in the areas surrounding the Lubombo Mountains [15]. Interventions took place primarily in Mozambique’s Maputo Province but were later extended to Gaza Province. The early termination of the LSDI in September 2010, and reduced IRS in Maputo Province thereafter, coincides with the increase observed in malaria cases in Maputo from 2011.

Metapopulation modelling is one method to describe movement between geographical areas with several applications in malaria and other infectious diseases. The metapopulation concept has been used to examine the spread of chloroquine resistance [16], model malaria transmission assuming the migration of the mosquitoes only [17–19], and account for human migration also [20–23]. Mathematical modelling of malaria in Mpumalanga includes a climate-based fuzzy distribution model [24], an eco-hydrological model [25] and the use of the SaTScan methodology to detect local malaria clusters to guide the Mpumalanga Malaria Control Programme [26]. The metapopulation model presented in this paper is developed to assess the impact of proposed elimination-focused policy interventions in Mpumalanga. This is the first study in Mpumalanga designed for this purpose and the first to do so in South Africa since the call for malaria elimination. The metapopulation structure is used to describe movement between five municipalities in the Ehlanzeni District on the eastern border of Mpumalanga and more importantly, movement between these municipalities and Maputo Province (MP), Mozambique. A stochastic, non-linear, ordinary-differential equation model fitted to the Mpumalanga and Maputo malaria case-notification data, is used to predict the impact of the following interventions (alone and in combination): scale-up of Vector Control, Mass Drug Administration (MDA), a Focal Screen and Treat (FSAT) campaign and foreign source reduction.

## Methods

### Ethics Statement

Ethical approval for use of the data was obtained from the Mpumalanga Department of Health and the University of Cape Town Human Research Ethics Committee.

### Transmission Model

**Fig 2:**
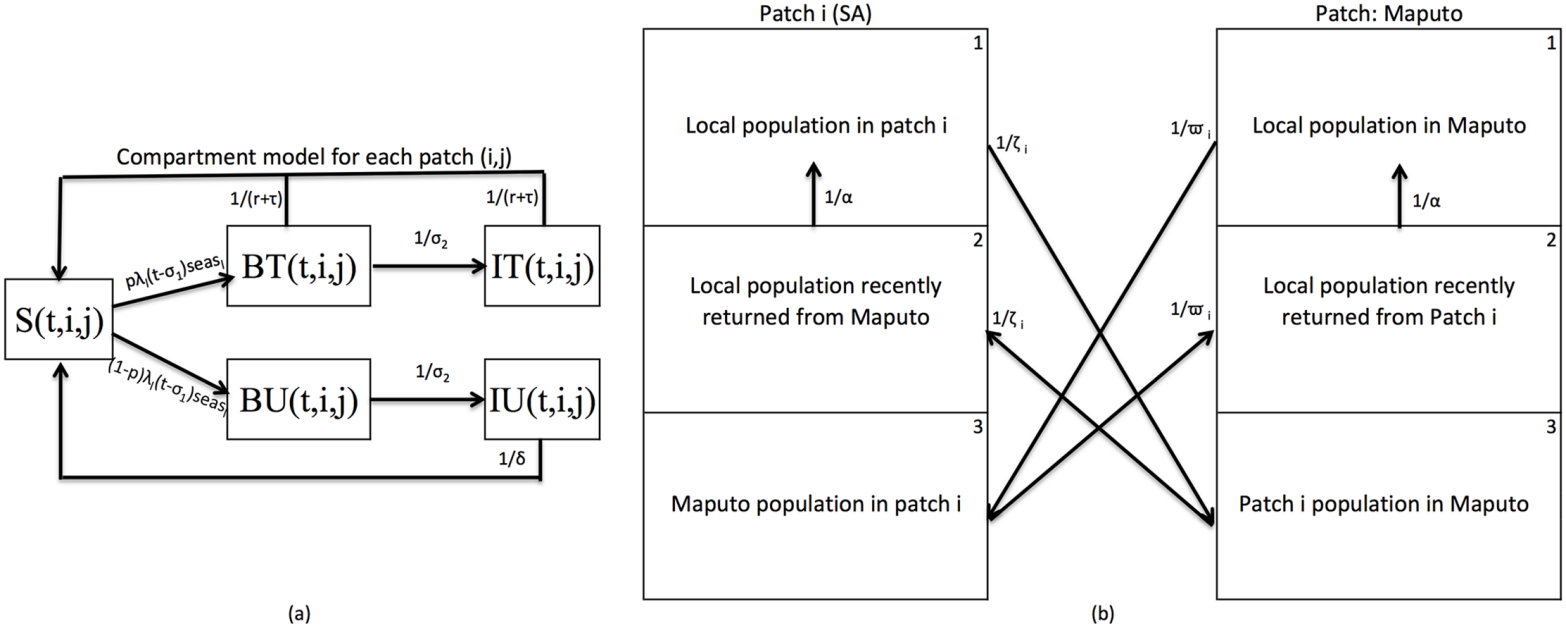
Metapopulation Model flow (a) Compartment transmission model for each patch i (1–6) with sub-patch j (1–3) at time step *t* with compartments S -Susceptible, BT—blood-stage and treated, BU—blood-stage and untreated, IT—Infectious and treated, and IU—Infectious, asymptomatic and untreated. (b) Metapopulation structure highlighting human movement between each local patch *i* *ϵ* {1, 2, 3, 4, 5} and foreign patch 6. Other parameters are described in S1 Text.

Metapopulation models divide a population into a number of discrete patches under the assumption that the sub-populations are well-mixed/homogenous. This structure allows for the modelling of transmission within and between different populations. The area under consideration is divided into six patches: five patches for the five municipalities in Ehlanzeni District (Thaba Chewu (TC), Mbombela (MB), Umjindi (UJ), Nkomazi (NK) and Bushbuckridge (BB)) and one patch for Maputo Province. Each patch is further divided into three sub-patches representing (1) the local population of the patch currently in the patch, (2) the local population of the patch having returned from travel to a foreign place (Maputo, if the patch is South African and vice versa) and (3) the population from the foreign place currently in the patch (Fig 2). In each sub-patch, the population is divided into five compartments representing the population susceptible to malaria (S), the population infected with asexual blood-stage malaria parasites (BT and BU) and the population at the infectious-stage (IT and IU) i.e. those carrying gametocytes (Fig 2a). The blood- and infectious-stage compartments are further stratified according to whether the infection is treated (T) or not (U) and the latent liver stage of the infection is incorporated as a delay in the flow between the susceptible and blood-stage compartments. Movement between compartments is governed by parameters described in Table 1. While the seasonal nature of transmission is incorporated in the model, the mosquito population is not modelled directly as it is assumed that the mosquito dynamics operate on a faster time-scale than the human dynamics. As such, the mosquito population can be considered to be at equilibrium with respect to changes in the human population [27]. Transmission from human to mosquito to human is thus incorporated through the parameter *β*, the number of infectious bites, multiplied by the proportion of infectious humans, delayed by four time steps. This delay takes accounts for life cycle of the mosquito from the first human bite to the second human bite.

**Table 1:** Values, descriptions and sources of the parameters driving the base metapopulation model of transmission. (*i* = {*TC*; *MB*; *UJ*; *NK*; *BB*; *MP*}) Values in parentheses are the assumed ranges for the parameter sensitivity analysis.

Parameter	Description	Value	Source
*N*	Population size for the six patches	2.5 × 10^6^	[52, 53]
*μ*	Mortality/birth Rate	$\frac{105}{10000}$	[43]
*δ*	Natural recovery period	26 weeks (24, 28)	[44–46]
*σ*_1_	Period between liver stage and blood-stage	7 days (5–10)	[47–49]
*σ*_2_	Period between blood-stage and onset of gametocytemia	2 weeks (1.8, 2.2)	[44, 50]
*r*	AL elimination half-life	6 days (4, 8)	[51]
*τ*	Time to seek treatment	1/2 week	Expert opinion
*p*	Proportion of local infected population receiving treatment	0.95	[35, 36]
*pf*_*yr*_	Proportion of foreign infected population that receive treatment in a local patch	*pf*_1_ = 0.5655(0.5652, 0.5658) (pre April 2005)*pf*_2_ = 0.5500 (0.5494, 0.5506) (post April 2005)	Estimated from model fitting process
*seas*_*i*_	Seasonal forcing function	Derived from data	[38]
*β*_*i*_	Annual number of mosquito bites per person x proportion of bites testing positive for sporozoites for patch *i*	*β*_*TC*_ = 0.334 (0.244, 0.425) *β*_*MB*_ = 2.178 (2.056, 2.300) *β*_*UJ*_ = 0.805 (0.700, 0.910) *β*_*NK*_ = 1.330 (1.310, 1.350) *β*_*BB*_ = 8.304 (7.903, 8.705) *β*_*MP*_ = 94.999 (93.327, 96.671)	Estimated from model fitting process
$\frac{1}{\alpha }$	Rate of movement between sub-patch 2 and sub-patch 1	2 weeks^−1^(1.75, 2.25)	Expert opinion
$\frac{1}{k}$	Rate of movement between 5 Mpumalanga municipalities	1/48.603 (1/51.328, 1/45.787) weeks^−1^	Estimated from model fitting process
$\frac{1}{v_{y}}$	Maputo residents: Rate of movement between Maputo and 5 Mpumalanga municipalities	$\frac{1}{v_{1}}=1/1258.828\;{\text{weeks}}^{-1}\left(1/1261.249,1/1256.407\right)$ (pre-April 2005) $\frac{1}{v_{2}}=1/319.042\;{\text{weeks}}^{-1}\left(1/322.796,1/315.333\right)$ (post April 2005)	Estimated from model fitting process
$\frac{1}{z}$	Mpumalanga residents: Rate of movement between 5 Mpumalanga municipalities and Maputo	$\frac{1}{z}=1/765.19\;{\text{weeks}}^{-1}$	Estimated from model fitting process
*fwgt*	Foreign movement weight intensity	8.385 (8.232, 8.537)	Estimated from model fitting process
*lwgt*	Local movement weight intensity	2.613 (2.607, 2.618)	Estimated from model fitting process
*vc*[*i*, *t*]	*vccov*[*i*, *t*] × *vef*		
*vccov*[*i*, *t*]	Vector Control Coverage	0.22–0.90	Derived from data
*vef*	Effectiveness of vector control	0.900 (0.897, 0.903)	Estimated from model fitting process

Human movements are incorporated in two ways. Local movements are allowed between the five Mpumalanga patches (from all five compartments in all three sub-patches). Foreign travel is allowed between the Maputo patch and any of the five Mpumalanga patches (from all five compartments) as illustrated in Fig 2b. It was not possible to access quality temporal data on human movement patterns between the six study areas. Thus a gravity model was considered to model human migration. Migration is modelled as a constant rate between patches that is inversely weighted by distance. This rate is varied stochastically in model. The constant rate is inferred to parameter estimation in the transmission model and a sensitivity analysis of this rate is conducted and presented in S1 Text.

### Data-fitting

The metapopulation model is fitted to weekly case-notification data of treated cases from Mpumalanga and Maputo from 2002 to 2008, and then cross-validated against the data from 2009 to 2012. In Southern Africa, most malaria transmission occurs during the summer rainfall season between October and May. Silal *et al*. (2013) describes in detail the characteristic triple peaked pattern in the Mpumalanga case data with peaks occurring during October, December/January and April/May while Maputo Province exhibited the December and April peaks only. Seasonal forcing, described as a function of rainfall, geography and source of infection, that determines the behaviour of transmission for the six patches is derived from the data using Seasonal decomposition of Time series by LOESS (STL) methods for extracting time series components [28]. ACT drug therapy and IRS implemented between 2002 and 2008 are included in the model.

The model is run deterministically from 1990 to reach a steady state before being fitted to data from 2002. The model-predicted weekly treated malaria cases are fitted to the data from 2002 to 2008 using the maximum likelihood approach by assuming an underlying Poisson distribution with rate *λ* as the number of treated cases per week. Several parameters are estimated through the data-fitting process using the particle swarm optimisation routine, a population-based global search algorithm [29, 30]. The model with the estimated parameter values is then run for a further three years and compared to data between 2009 and 2012. A full description of the data-fitting method is presented in S1 Text. Model development, fitting and subsequent analysis was performed in R v3.02 [31]. The particle swarm optimisation routine was performed using the R package hydroPSO v0.3-3 [32, 33].

### Interventions

The interventions to be tested include: scaling-up of vector control from current levels, mass drug administration in three local patches, a focal screen and treat campaign at the Mpumalanga-Maputo border, foreign source reduction through vector control and MDA in Maputo Province only and relaxing vector control during the FSAT campaign. The scale up of vector control is assumed to occur uniformly within each patch (if vector control is being performed in the patch). MDA is modelled at a coverage below 100%. Being a metapopulation model, individuals are not tracked, therefore all members of a metapopulation are equally likely to receive MDA, though no member can receive it twice in the same round. With FSAT, all border-crossers are equally likely to receive FSAT, and they are tracked as a group until such time as any future infection will be a locally sourced one. Source reduction in Maputo Province is conducted at the metapopulation level only. Thus it is assumed that the vector control and MDA are performed uniformly throughout the province. A full description of the interventions modelled can be found in S1 Text.

## Results

### Estimation of Parameters through data-fitting

**Fig 3:**
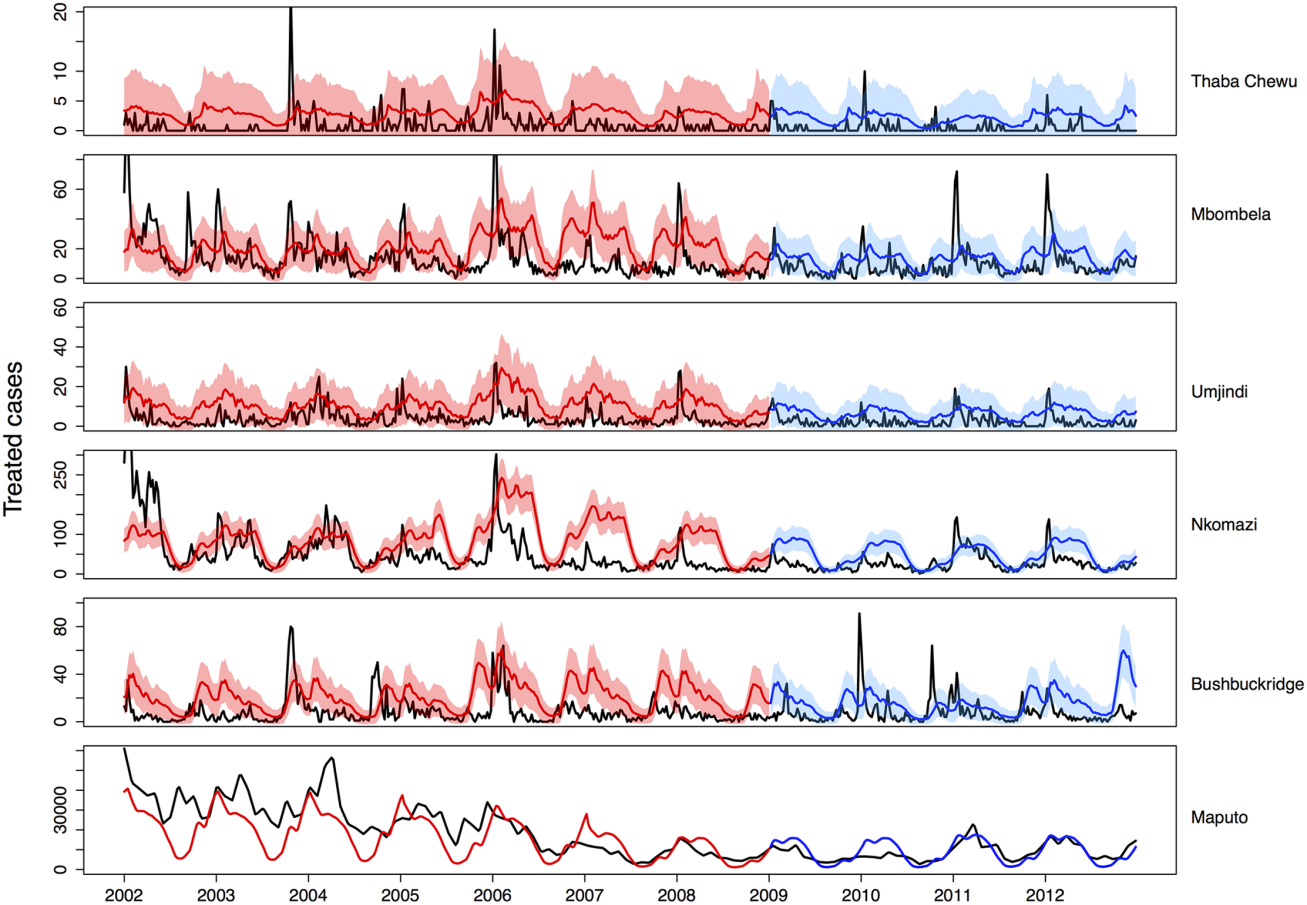
Predicted average weekly treated cases (blue: 2002–2008 red: 2009–2012) fitted to and validated with data (black). The 95% uncertainty range for weekly case predictions is shown.

Fig 3 shows the notified case data for Maputo and the five municipalities (black) with the model output from the fitting process (red) and the predicted model output for 2009 to 2012 (blue). As the data was fitted to each sub-patch simultaneously, Fig 3 represents only a summation of the data-fitting. More detailed output on the data-fitting is available in S1 Text. The parameters driving the model and those estimated through data-fitting procedures are presented in Table 1.

The proportion of infections treated varies widely across Africa with some estimates as low at 10% and others as high as 90% [34]. In South Africa and Mozambique, the proportion of the local infected population receiving treatment was informed by two studies [35, 36]. Castillo-Riquelme *et al*. (2008) conducted household surveys in Mozambique and South Africa between 2001 and 2002 to evaluate malaria-related treatment-seeking behaviour and found that 100% of respondents in Mpumalanga and 99% of respondents in Mozambique with recent malaria sought treatment. Hlongwana *et al*. (2011) conducted a study on malaria-related knowledge and practices in Bushbuckridge Municipality in 2008 and reported that 99% of respondents would seek malaria treatment (95% Confidence interval: 97.5- 99.5%). Different rates are fitted for the foreign treatment proportion and foreign movement rate before and after April 2005 when the South African and Mozambican governments waived short-stay visa requirements which subsequently led to increased movement between the two countries [37].

### Interventions

Interventions are tested on a stochastic version of the fitted model; the same intervention is applied to multiple model runs such that its impact on local infections can be described with a mean effect and a 95% confidence interval. Stochastic uncertainty and parameter sensitivity has been accounted for as follows. The model is run stochastically by treating each flow between compartments at each time point *t* as a random realisation of a Poisson process with rate *λ*, the deterministic flow value at that time, and by simulating the parameter values from their 95% confidence intervals. Eight random seeds were selected per parameter set and 150 parameter sets were simulated. The parameters were simulated from uniform distributions with ranges in parentheses in Table 1. In line with the 2018 goal, elimination is defined as sustained zero local infections (treated and untreated). Thus imported infections may still occur, though with zero secondary cases. A summary of key findings may be found in Box 1.

#### Further Scaling up Vector Control

Vector control in Nkomazi and Bushbuckridge municipalities is achieved through high-coverage IRS with larviciding at selected sites. Vector control is used less intensively in Mbombela and Umjindi municipalities and is not currently conducted in Thaba Chewu municipality. Assuming vector control remains at 2012 levels until 2020, the impact of a scale-up in vector control is modelled as a percentage decrease in *β*_*i*_, the number of local human contacts with infectious mosquitos. Scaling up vector control in Nkomazi, Bushbuckridge and Mbombela municipalities i.e. decreasing *β*_*i*_ by a further 10%, is predicted to result in a decrease in local infections in all municipalities (Fig 4.1). This includes Umjindi where vector control was not scaled-up and Thaba Chewu where vector control was not conducted at all. The decrease, though substantial in some municipalities, is not predicted to be enough to eliminate local malaria owing to the continued flow of imported infections into the province. While onward transmission from imported cases may be decreased by vector control, the inflow of imported cases is otherwise unhampered by increased vector control in the province. The seasonal decomposition of local cases suggests that they occur earlier in the season in Bushbuckridge municipality than the other municipalities [38]. Exploring a scenario of scaling up vector control first in Bushbuckridge followed by Nkomazi municipality is predicted to result in further decreases in local infections in the deterministic model, but these decreases were trivial in the stochastic model.

**Fig 4:**
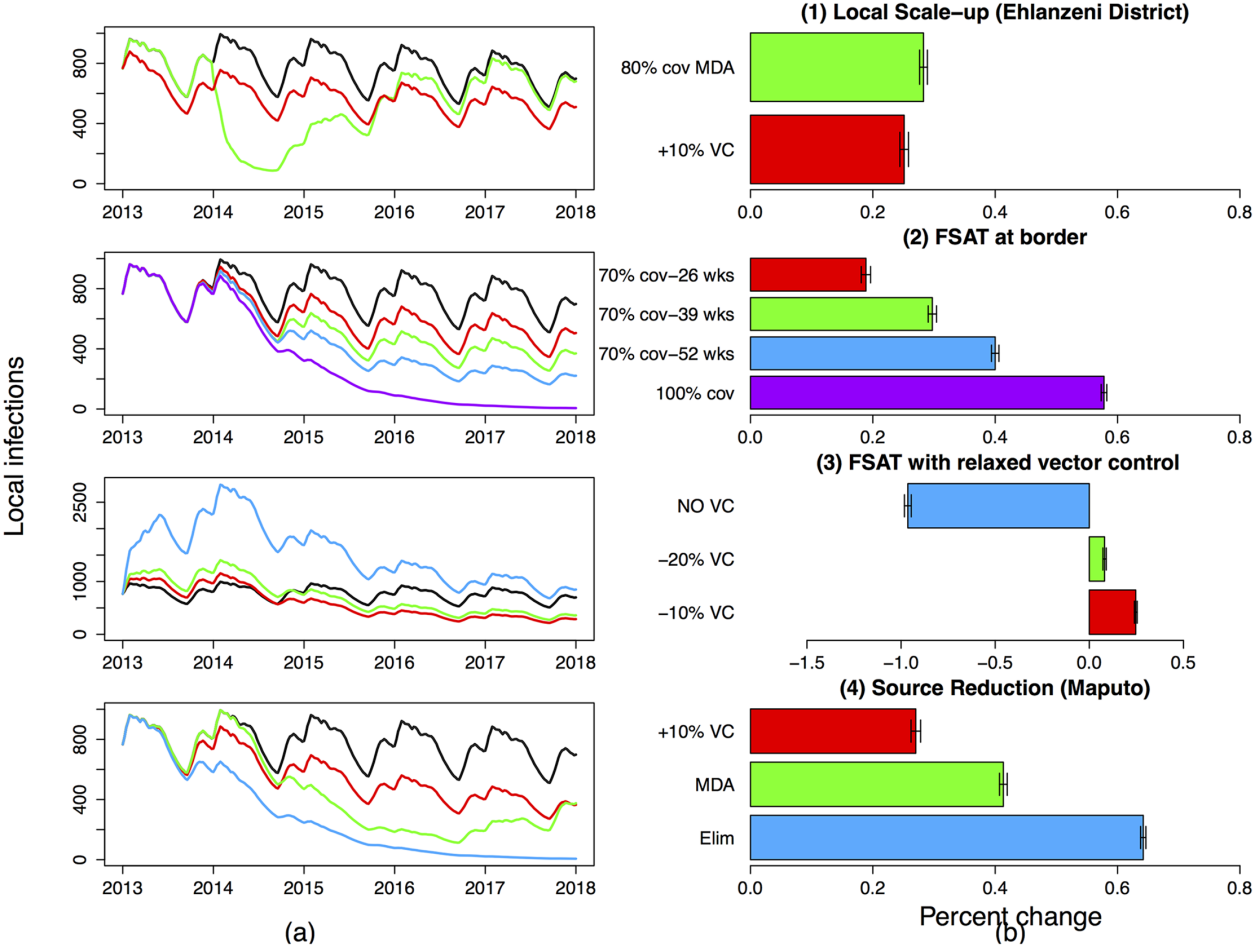
Predicted impact of interventions on the number of local infections in the Ehlanzeni district (summation of the five local patches). (a) shows the impact of the interventions on local infections in Ehlanzeni district through time compared to the base case of no interventions (black) and (b) shows the percentage change (increase or decrease) in point estimates of local infections due to the interventions between 2013 and 2018. (1) Local Scale-up: Increase in local vector control so as to reduce the mosquito-human contact rate by a further 10% (red), three consecutive two-monthly rounds of MDA in Mbombela, Nkomazi and Bushbuckridge Municipalities (green). (2) FSAT at the border: at 70% coverage for 26 weeks (red), 39 weeks (green), 52 weeks (blue) and 52 weeks at 100% coverage (purple). (3) Reducing Vector Control: FSAT at the border at 70% coverage administered all year round while simultaneously reducing vector control by 10% (red), 20% (green) and stopping vector control altogether (blue). (4) Source Reduction: 10% scale up of vector control in Maputo (red), three consecutive two-monthly rounds of MDA in Maputo (green) and eliminating malaria in Maputo (blue). The base case of no intervention is shown in black.

#### Mass Drug Administration

Mass Drug Administration is an intervention aimed at treating all individuals without screening and regardless of disease status. Though MDA may be targeted at certain populations, it is still improbable that every member of the population receives treatment and thus MDA is modelled with a coverage rate below 100%. The model predicts that when MDA coverage achieves 80% coverage for each of three consecutive rounds of two month intervals in Nkomazi, Bushbuckridge and Mbombela municipalities, local infections decrease substantially in all five municipalities, though this decrease is not predicted to eliminate local malaria (Fig 4.1). While this predicted decrease is substantial, it is short-lived with infections predicted to reach previous levels within three years. As MDA is administered regardless of the source of infection, foreign infections in the five municipalities are also predicted to decrease substantially during MDA but revert back to previous levels within 18 months of the end of the MDA as the subsequent inflow of imported infections remains unaffected.

#### Focused Screen and Treat Campaign at the Mpumalanga-Maputo Province border post

It is a resource-intensive exercise to treat all individuals regardless of disease status. Screen and Treat campaigns include an additional stage of screening individuals resulting in only those testing positive receiving treatment. However, these campaigns are unlikely to achieve very high coverage in large target populations. A high-coverage Screen and Treat campaign is more feasible if focused on a subset of the population only.

The FSAT campaign is modelled at a border entry point between Mpumalanga and Maputo. Thus the proportion of the population targeted in this intervention is substantially lower than the MDA campaign previously modelled, where three municipalities were targeted. Fig 4.2 shows the predicted impact of administering FSAT (at different coverage rates and for different durations) at the border entry point. The rationale for screening travellers and treating positive cases before entry into Mpumalanga is that imported infections now comprise the majority of Mpumalangas malaria cases, with Mozambique being the most frequent source of infection. The advantage of the metapopulation structure of the model is that the impact of FSAT is modelled on both the foreign population entering Mpumalanga and the local population returning from travel to Maputo Province. FSAT is modelled at a 70% coverage rate to account for limitations in test sensitivity, illegal border crossing, and other forms of entry into the province. Vector control activities are assumed to continue at 2012 levels. Fig 4.2 shows the predicted impact of different FSAT schemes at the border on *local* infections i.e. the effect on onward transmission as result of fewer imported cases in the Mpumalanga patches. In particular FSAT is modelled at 70% coverage for six months from October to March (red), nine months from September to May (green), all year round (blue) and all year round at 100% coverage (purple) compared to no FSAT (black). The impact on local infections is predicted to be substantial regardless of the duration of FSAT. At 70% coverage, FSAT is predicted to be most effective when performed throughout the year (blue) as even a few imported cases over the non-malaria season can maintain local transmission. Even when performed throughout the year at 70% coverage, FSAT is insufficient to eliminate local infection. Elimination is predicted if FSAT is performed continuously throughout the year at 100% coverage. This is of course unrealistic but serves to illustrate the prediction that if imported infections are treated before entry into Mpumalanga (as opposed to being prevented altogether), elimination of local malaria becomes a possibility. Like MDA, FSAT has a short-lived benefit as the model predicts a reversion to previous levels approximately three years after the end of the intervention.

#### FSAT with Relaxed Vector Control

Focused Screen and treat campaigns have been predicted to be effective in reducing infections substantially if sustained for a long period. In dedicating resources to this intervention, it may be tempting to relax the vector control effort in an attempt to shift resources rather than procure additional resources. Fig 4.3 shows the impact of an FSAT campaign on the border between Maputo and Mpumalanga, at 70% coverage, administered all year round while simultaneously relaxing the Mpumalanga vector control effort. The black line represents the model prediction if the only intervention is vector control at current levels. FSAT is then modelled while simultaneously relaxing vector control by 10% (red), 20% (green) and stopping vector control all together (blue). The model predicts that the impact of a sustained FSAT programme is dampened by a reduction in vector control with the impact of FSAT being zero and local infections increasing substantially, if vector control is abandoned all together.

#### Source Reduction

In 2012, 87% of all reported malaria cases in Mpumalanga were imported, and Fig 4.2 shows that using FSAT to treat imported cases is predicted to decrease local infections substantially. Therefore, source reduction is explored by assessing the effects of scaling up vector control and MDA in Maputo Province. Fig 4.4 shows the predicted impact of a scale up of vector control in Maputo Province such that the local human-infectious mosquito contact rate is decreased by 10% (red), three consecutive two-monthly rounds of MDA in Maputo Province (green) and eliminating malaria in Maputo Province (blue), on local infections in Mpumalanga. The scale-up in vector control in Maputo Province is predicted to have a delayed but substantial impact on local infections in Mpumalanga through the decrease in the number of imported infections. Likewise, the impact of six months of MDA in Maputo Province is also predicted to decrease local infections substantially, though predicted local infections revert slowly to previous levels once the MDA in Maputo Province has stopped. If malaria is eventually eliminated in Maputo Province (blue) and vector control is continued in Mpumalanga at 2012 levels, the model predicts that local malaria will also be eliminated in Mpumalanga. This prediction is in line with the model prediction that treating all imported infections will also lead to the elimination of local infection in Fig 4.2.

**Box 1: Highlights of model findings**

The stochastic metapopulation transmission model developed to simulate transmission in Ehlanzeni District, Mpumalanga, and Maputo Province, Mozambique has made predictions that lead to the following conclusions:

- Scaling-up vector control will decrease prevalence but not eliminate malaria in the presence of imported infections.
- Mass interventions lead to large and immediate decreases in prevalence but will result in a rebound in prevalence three years after the intervention has stopped.
- Smaller scale interventions such as FSAT at the border have the same large but short-lived impact of mass interventions and are most effective if conducted throughout the year as the presence of even a few imported infections, leads to onward transmission.
- Reducing vector control in favour of FSAT dampens the impact of FSAT in both the short and long terms.
- Source reduction is likely to be effective in decreasing local prevalence, whether through better control or elimination at the source.
- There is no “one size fits all” strategy to achieve malaria elimination and a tailored approach is needed to address linkages between populations. For example, in the case of Mpumalanga province, the high level of imported infections suggests that a regional approach to malaria elimination will be more successful than a nationally focused one.

## Discussion

South Africa aims to achieve malaria elimination by 2018. A malaria-elimination strategy should aim to interrupt the transmission cycle and prevent it from being reestablished. An elimination strategy that employs a ‘more of the same’ approach may decrease the malaria burden, but will be insufficient to eliminate it as the focus needs to shift from better overall control to the identification of residual transmission foci leading to the last few infections. The interruption of the transmission cycle and prevention of its reestablishment theoretically requires three elements: (1) the elimination of the mosquito vector to prevent onward transmission, (2) inhibiting the inflow of imported infections and (3) reduction of infections at their source [39]. The first element is operationally unfeasible and has not been recommended [40]. The second element could be achieved if borders were closed, or more realistically if imported infections were identified and treated at border entry points before they can contribute to the infectious reservoir locally. The third element would require regional collaboration with these sources of imported infections to reduce transmission in the region [39]. South Africa has employed consistent IRS and artemisinin-based combination therapy to control malaria. This paper has explored a range of additional interventions (scale-up of vector control, MDA, FSAT and source reduction) that speak to all three key elements of elimination.

Mass Drug Administration is an intervention that is resource intensive in terms of labour and the cost of drugs. It is also an intervention that needs to be acted out quickly and efficiently to achieve desired target coverage rates. In a single patch deterministic model of malaria in Mpumalanga, Silal *et al*. (2014) predicted that in the absence of imported infections, MDA applied continuously over six months at 80% coverage would be sufficient to eliminate local malaria, but even repeated annual rounds of MDA for seven years is insufficient to eliminate local malaria at the current level of imported infections because MDA does not interrupt the inflow of imported infections. The stochastic metapopulation model presented in this paper also predicts that MDA applied in the three municipalities with the highest incidence has a large impact but this is short-lived because there is no impact on the flow of imported infections. A Focused Screen and Treat campaign focused on treating infections at the border control point between Maputo Province and Mpumalanga is also predicted to have a large impact, but is not enough to eliminate local malaria on its own unless the unlikely scenario of all imported infections being screened and treated is achieved. As soon as the FSAT campaign is stopped, infections revert to previous levels within three years. This suggests that screening and treating infections at the border would need to be intense indefinitely (in the absence of new interventions) to minimise the impact of imported infections on malaria transmission.

In Mpumalanga, vector control has been conducted using high-coverage IRS with dichorodiphenyltrichloroethane (DDT) and larviciding at identified breeding sites. Scaling-up vector control as an elimination intervention may include intensifying the already extensive spraying programme, increasing the distribution of insecticide-treated bednets and the identification and larviciding of additional breeding sites. The purpose of a scale-up in vector control would be to decrease the potential for onward local transmission, though it is impossible to reduce this to zero. While effective if the majority of infections are locally sourced, the model predicts that increasing vector control alone will not eliminate local infection if the stream of imported infections is left unchecked. These predictions are in line with the single patch model in Silal *et al*. (2014) but the metapopulation model has predicted that increasing vector control in Nkomazi, Mbombela and Bushbuckridge municipalities only also leads to a knock-on decrease in malaria cases in the other two municipalities. Another recent modelling study found that at low receptivity levels, case management alone could not reliably prevent the reestablishment of transmission in the presence of medium to high importation rates [41].

While scaling-up vector control alone in Mpumalanga may not be enough to interrupt transmission and prevent its reestablishment, vector control at the source of imported cases has a large effect. The model predicts that if vector control is continued at current levels in Mpumalanga, but is scaled up in Maputo, the related decrease in local infections in Mpumalanga will be substantial. This decrease results because a smaller proportion of the population that travels into Mpumalanga will be infected and hence the infectious reservoir in Mpumalanga is reduced. These knock-on decreases in local infections in Mpumalanga are also predicted if MDA is performed in Maputo Province, although infections revert to previous levels a few years after the end of the MDA campaign. The model also predicts that if malaria were to be eliminated in Maputo, malaria would also be eliminated in Mpumalanga. These predictions highlight the need for and pivotal importance of cross-border/regional collaboration. The Lubombo Spatial Development Initiative, a trilateral agreement between South Africa, Mozambique and Swaziland, was initiated in 1999 and successfully reduced malaria cases by 78 to 95% in the border areas of South Africa and Swaziland within five years of the start of IRS and then ACT deployment in Maputo Province [15]. In September 2010, the earlier than expected ending of LSDI support for IRS (when the Global Fund withdrew support) resulted in sub-optimal spraying in Maputo and Gaza provinces. This coincides with the increase in malaria cases in Maputo Province from 2011 [14]. In allocating resources towards elimination-focused interventions, programme managers may wish to decrease routine activities to shift budgets towards elimination. It is important to remember that even in areas of very low transmission intensity (as seen in the pre-elimination phase), imported infections will augment the infectious reservoir, and since the vector remains present, imported infections may lead to onward transmission to the local population and a resurgence of malaria. The model predicted that the impact of FSAT could be dampened and even reduced to zero if current vector control efforts are reduced or stopped.

Varying levels of population immunity in Mozambique can impact results where decreases in immunity may lead to an increase in symptomatic cases or a greater proportion of infections are treated in South Africa. This could result in a reduction in the number of secondary cases if these infections are treated routinely. The impact of mass interventions is unaffected by varying population immunity in Mozambique as interventions are deployed *enmasse* through drug distribution or screening in the case of FSAT.

This paper presents the findings of a stochastic, metapopulation, non-linear, differential equation model of five municipalities in Mpumalanga, South Africa and in neighbouring Maputo Province, Mozambique. While the current metapopulation structure allows for more disaggregated modelling than the model presented in Silal *et al*. (2014), mass interventions if administered, will most likely be performed in smaller hotspot areas within a municipality and may be more accurately modelled if patches are disaggregated further or agent-based modelling is used to incorporate heterogenous behaviour among individuals. This metapopulation comprised six patches and while this methodology may theoretically be extended to any number of patches, several aspects must be taken into account. Computationally, extending the methodology to a large number of patches *n* with $\frac{n(n-1)}{2}$ links between patches (499500 in the case of 1000 patches) will result in the equations becoming too numerous to be efficient. Further, depending on the size of the populations of interest and the available data, one could consider either agent-based modelling (disaggregating populations into individuals) or a simplified approach where for example, patches are linked using weights rather than flows. Disaggregating populations increases the data requirements of the model including details on population movement between patches. This data is often not available or unreliable for larger administrative areas such as those defined as patches in this model. Future work includes incorporating an economic cost component to the model, exploring the impact of border control through FSAT in greater detail, particularly around issues of implementation, and incorporating vector population dynamics in the model so that vector control activities such as indoor residual spraying and larviciding may be modelled explicitly and thereby allow for an exploration of post-elimination maintenance strategies to detect outbreaks and prevent the resurgence of local transmission.

## Conclusion

To eliminate malaria by 2018, the government of South Africa will need to design and implement an elimination strategy tailored for a country with a high level of imported infections. A regionally focused strategy may stand a better chance at achieving elimination in Mpumalanga and South Africa compared to a nationally focused one in the face of frequent population movement between the pre-elimination area and neighbouring high transmission intensity regions [42]. Mathematical modelling has been used in this paper to test out elimination-focused strategies like scaled up vector control, MDA, FSAT at the border and foreign source reduction). In this manner, mathematical modelling may be used to inform government policy to tailor a strategy that captures the malaria situation not just in South Africa, but also in the immediate region, in order to inform feasible strategies to enable malaria elimination in the foreseeable future.

## Supporting Information

S1 Text This file contains the model description, model equations and additional information on the data-fitting process. (PDF)
