# Correction: Hitting a Moving Target: A Model for Malaria Elimination in the Presence of Population Movement

**DOI:** 10.1371/journal.pone.0159784

**Published:** 2016-07-14

**Authors:** Sheetal Prakash Silal, Francesca Little, Karen Irma Barnes, Lisa Jane White

The reference Silal *et al*. (2014), which is cited three times in the Discussion, is omitted from the References list. The reference is: Silal, S. P., Little, F., Barnes, K. I., & White, L. J. (2014) Towards malaria elimination in Mpumalanga, South Africa: a population-level mathematical modelling approach. Malaria Journal, 3:297.

The caption of Fig 1 is incomplete in the published article. Please see Fig 1 and its complete caption here.

**Fig 1:**
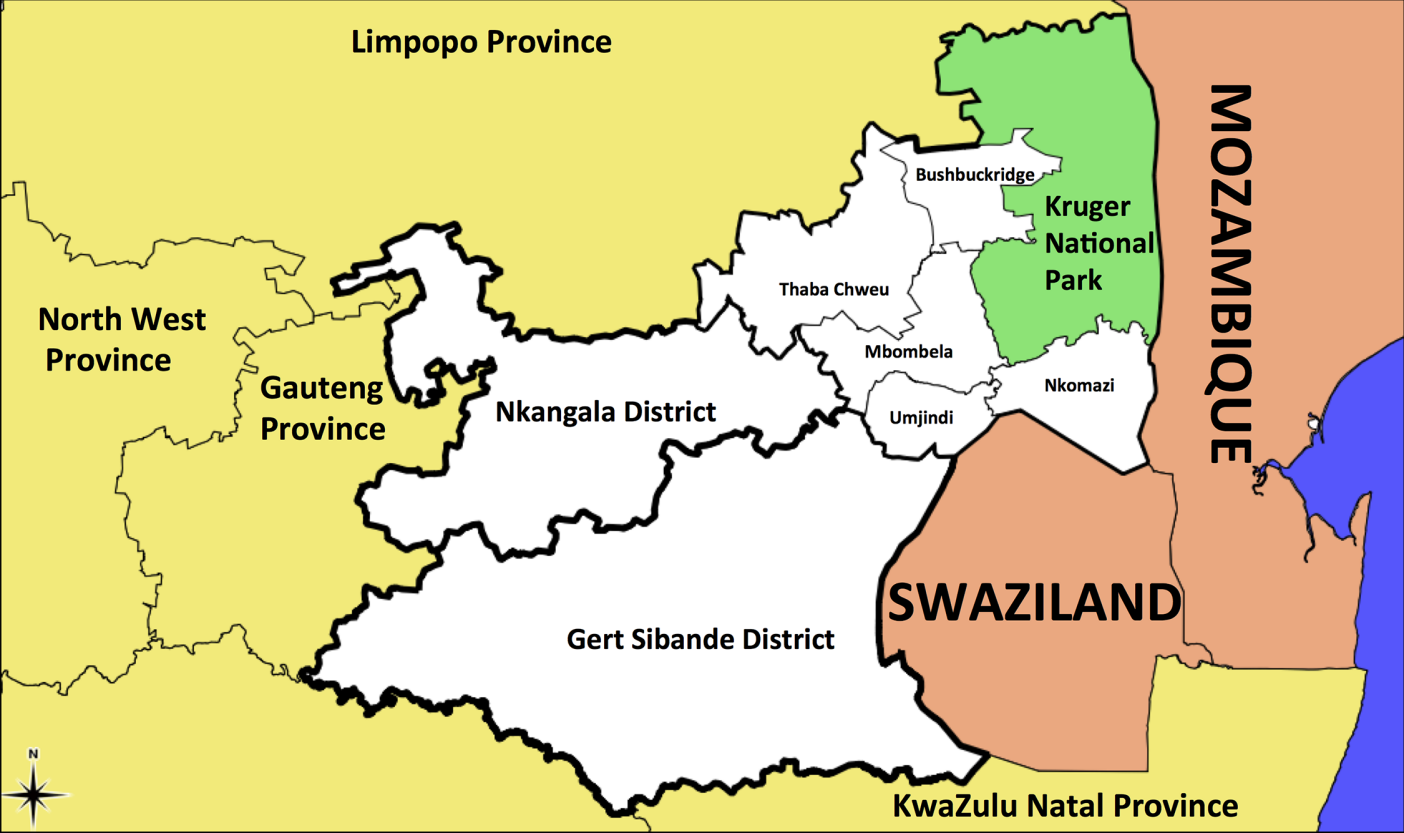
A map of Mpumalanga Province in relation to Mozambique and Swaziland (Source: Mpumalanga Malaria Elimination Programme). This figure was previously published in Silal SP, Little F, Barnes KI, White LJ (2015) Predicting the impact of border control on malaria transmission: a simulated focal screen and treat campaign. Malaria Journal 14:268. DOI: 10.1186/s12936-015-0776-2.
