# Supplementary material for: Hitting a Moving Target: A Model for Malaria Elimination in the Presence of Population Movement
Source: PLoS One. 2015 Dec 21;10(12):e0144990. doi: 10.1371/journal.pone.0144990 (PMC4686217; doi:10.1371/journal.pone.0144990)
Supplement: S1 Text — (PDF) [file pone.0144990.s001.pdf]

Hitting a moving target: a model for malaria elimination in the presence of population movement

Supplementary File 1: Mathematical Model Description

Sheetal Prakash Silal *[1], Francesca Little [1] , Karen Irma Barnes [2] and Lisa Jane White [3,4]

[1]Department of Statistical Sciences, University of Cape Town, Cape Town, South Africa
[2]Division of Clinical Pharmacology, Department of Medicine, University of Cape Town, Cape Town, South Africa
[3]Mahidol-Oxford Tropical Medicine Research Unit, Mahidol University, Bangkok, Thailand
[4]Centre for Tropical Medicine, Nuffield Department of Clinical Medicine, Churchill Hospital, University of Oxford, Oxford, UK

Email: Sheetal Prakash Silal *- sheetal.silal@uct.ac.za; Francesca Little - francesca.little@uct.ac.za; Karen Irma Barnes - karen.barnes@uct.ac.za; Lisa J White - lisa@tropmedres.ac;

*Corresponding author

Summary Equations: Metapopulation Model of Transmission

The model comprises six patches; five to represent Mpumalanga municipalities, and one for Maputo province, Mozambique. Each patch comprises three sub-patches: (1) the local population of patch i currently in patch i, (2) the local population of patch i having returned from travel to a foreign place (Maputo, if the patch is South African and vice versa) and (3) the population from the foreign place currently in patch i. In each sub-patch, the population is divided into five compartments representing the population susceptible to malaria (S), the population infected with malaria at the asexual blood-stage (B) and the population at the infectious stage (I). The blood and infectious stage compartments are further stratified according to whether the infection is treated (T) or not (U).

This system is described by a set of non-linear differential equations for which the compartment and parameter descriptions are given in Tables 1 and 2 respectively.

The force of infection $\lambda_i[t]$ for each patch i is a function of the level of vector control, the annual number of mosquito bites per person x proportion of bites testing positive for sporozoites for patch i (β_i) and the proportion of infectiousness in the population lagged to reflect the infectiousness proportion at the time the mosquito was infected.

$$\lambda_i[t] = (1 - vc_i[t] * vef)\beta_i \times \frac{IT_{i,1}[t-4] + IU_{i,1}[t-4] + IT_{i,2}[t-4] + IU_{i,2}[t-4] + IT_{i,3}[t-4] + IU_{i,3}[t-4]}{N_i}.$$

Population migration between the different patches is characterised by three sets of movements:

1. Movement may occur between any two of the five Mpumalanga patches i and j at a rate $\frac{1}{\kappa_{i,j}}$ where

$$\frac{1}{\kappa_{i,j}} = \frac{1}{k} \times \frac{\frac{1}{(1+\sqrt{(x_i-x_j)^2+(y_i-y_j)^2})^{lwgt}}}{\sum_{i=1}^{5} \frac{1}{(1+\sqrt{(x_i-x_j)^2+(y_i-y_j)^2})^{lwgt}}} \qquad \text{if } i,j = 1,2,3,4,5 \ \& \ i \neq j$$
$$= 0 \qquad \text{if } i \text{ or } j = 6 \text{ (local movement only)}$$
$$\frac{1}{\kappa_{i,j}} = \frac{1}{\kappa_{j,i}}$$

where (x_i, y_i) and (x_j, y_j) are the centroid coordinates for patches i and j respectively. This movement is weighted inversely by distance so that movement between South African patches that are closer together occurs at a higher rate than those further apart. The parameter $lwgt$ determines the degree of linkage between patches i and j.

2. Movement may occur when South African citizens cross the border into Maputo (from patch $i = 1-5$ in sub-patch 1 to patch 6 in sub-patch 3) and return (patch 6 in sub-patch 3 to patch $i = 1-5$ in sub-patch 2) at a rate of $\frac{1}{\zeta_{i,6}}$ where

$$\frac{1}{\zeta_{i,6}} = \frac{1}{z} \times \frac{\frac{1}{(1+\sqrt{(x_i-x_6)^2+(y_i-y_6)^2})^{fwgt}}}{\sum_{i=1}^{5} \frac{1}{(1+\sqrt{(x_i-x_6)^2+(y_i-y_6)^2})^{fwgt}}} \qquad \text{if } i = 1,2,3,4,5$$
$$= 0 \qquad \text{otherwise}$$
$$\frac{1}{\zeta_{i,6}} = \frac{1}{\zeta_{6,i}}$$

where (x_i, y_i) and (x_6, y_6) are the centroid coordinates for patches i and 6 respectively. This movement is weighted inversely by distance so that movement between the South African patches and Maputo that are closer together occurs at a higher rate than those further apart. The parameter $fwgt$ determines the degree of linkage between patches i and j.

3. Movement may also occur when Mozambican citizens cross the border into Mpumalanga (from patch 6 in sub-patch 1 to patch $j = 1-5$ in sub-patch 3) and return (patch $j = 1-5$ in sub-patch 3 to patch 6 in sub-patch 2) at a rate of $\frac{1}{\varpi_{6,j}}$ where

$$\frac{1}{\varpi_{6,j}} = \frac{1}{v_{yr}} \times \frac{\frac{1}{(1+\sqrt{(x_6-x_j)^2+(y_6-y_j)^2})^{fwgt}}}{\sum_{i=1}^{5} \frac{1}{(1+\sqrt{(x_6-x_j)^2+(y_6-y_j)^2})^{fwgt}}} \qquad \text{if } j = 1,2,3,4,5 \ \&\ yr = 1,2$$

$$= 0 \qquad \text{otherwise}$$

$$\frac{1}{\varpi_{6,j}} = \frac{1}{\varpi_{j,6}}$$

where (x_6, y_6) and (x_j, y_j) are the centroid coordinates for patches 6 and j respectively. This movement is weighted inversely by distance so that movement between the South African patches and Maputo that are closer together occurs at a higher rate than those further apart. The parameter $fwgt$ determines the degree of linkage between patches i and j.

This leads to the following set of differential equations. Footnotes have been added where needed to describe the flows between compartments. Footnotes appear at the first occurrence of each type of flow.

Sub-patch 1 (Local population): For each patch i moving to patch j $(i, j \ \epsilon \{1, 2, .., 6\}, j \neq i)$:

$$\frac{dS_{i,1}}{dt} = \underbrace{\mu N_i}_{(1)} - \underbrace{\lambda_i[t-\sigma_1]seas_i[t]S_{i,1}}_{(2)} + \underbrace{\frac{1}{r+\tau}(BT_{i,1}+IT_{i,1})}_{(3)} + \underbrace{\frac{1}{\delta}IU_{i,1}}_{(4)} + \underbrace{\frac{1}{\alpha}S_{i,2}}_{(5)} + \underbrace{\sum_j \frac{1}{\kappa_{i,j}}(S_{j,1}-S_{i,1})}_{(6)}$$
$$- \underbrace{\frac{1}{\varpi_{i,j}}S_{i,1}}_{(7)} - \underbrace{\frac{1}{\zeta_{i,6}}S_{i,1}}_{(8)} - \underbrace{\mu S_{i,1}}_{(9)}$$

$$\frac{dBT_{i,1}}{dt} = \underbrace{p\lambda_i[t-\sigma]seas_i[t](S_{i,1}+S_{i,2})}_{(10)} - \underbrace{\frac{1}{\sigma_2}BT_{i,1}}_{(11)} - \frac{1}{r+\tau}BT_{i,1} + \frac{1}{\alpha}BT_{i,2} + \sum_j \frac{1}{\kappa_{i,j}}(BT_{j,1}-BT_{i,1})$$
$$- \frac{1}{\varpi_{i,j}}BT_{i,1} - \frac{1}{\zeta_{i,6}}BT_{i,1} - \mu BT_{i,1}$$

$$\frac{dIT_{i,1}}{dt} = \frac{1}{\sigma_2}BT_{i,1} - \frac{1}{r+\tau}IT_{i,1} + \frac{1}{\alpha}IT_{i,2} + \sum_j \frac{1}{\kappa_{i,j}}(IT_{j,1}-IT_{i,1}) - \frac{1}{\varpi_{i,j}}IT_{i,1} - \frac{1}{\zeta_{i,6}}IT_{i,1} - \mu IT_{i,1}$$

$$\frac{dBU_{i,1}}{dt} = \underbrace{(1-p)\lambda_i[t-\sigma_1]seas_i[t](S_{i,1}+S_{i,2})}_{(12)} - \frac{1}{\sigma_2}BU_{i,1} + \frac{1}{\alpha}BU_{i,2} + \sum_j \frac{1}{\kappa_{i,j}}(BU_{j,1}-BU_{i,1})$$
$$- \frac{1}{\varpi_{i,j}}BU_{i,1} - \frac{1}{\zeta_{i,6}}BU_{i,1} - \mu BU_{i,1}$$

$$\frac{dIU_{i,1}}{dt} = \frac{1}{\sigma_2}BU_{i,1} - \frac{1}{\delta}IU_{i,1} + \frac{1}{\alpha}IU_{i,2} + \sum_j \frac{1}{\kappa_{i,j}}(IU_{j,1}-IU_{i,1}) - \frac{1}{\varpi_{i,j}}IU_{i,1} - \frac{1}{\zeta_{i,6}}IU_{i,1} - \mu IU_{i,1}$$

(1) Births in patch i

(2) Local incidence arising from sub-patch 1

(3) Recovery of treated blood and infectious stage infections at a rate dependent on the time to seek treatment and the time to recovery

(4) Recovery of untreated infections at a rate dependent on the duration of natural recovery

(5) Assimilation of population in sub-patch 2 (locals having returned from foreign travel) back into sub-patch 1 from whence they originated.

(6) Movement between local patches (1-5) out of and into the compartment

(7) Movement of local patch i population to foreign patch j when i=6 and j = 1-5; =0 for all other values of i as this rate is particular to movement of Maputo population (patch 6)

(8) Movement of local patch i population to foreign patch 6 when i=1-5; =0 for i=6 as this rate is particular to movement of the Mpumalanga population to and from Maputo (patches 1-5)

(9) Deaths in patch i from this compartment

(10) New infections destined to be treated having arisen from susceptible populations in sub-patch 1 (local population) and sub-patch 2 (local population having returned from foreign travel) as these are infections due to local transmission.

(11) Development of infectiousness at a rate dependent on the duration of the blood stage

(12) New infections destined to remain untreated (having arisen from susceptible populations in sub-patch 1 (local population) and sub-patch 2 (local population having returned from foreign travel) as these are infections due to local transmission.

Sub-patch 2 (Local population returning from foreign travel) : For each patch i moving to patch j $(i, j \epsilon \{1, 2, .., 6\}, j \neq i)$

$$\frac{dS_{i,2}}{dt} = -\underbrace{\lambda_i[t-\sigma_1]seas_i[t]S_{i,2}}_{(13)} + \frac{1}{r+\tau}(BT_{i,2} + IT_{i,2}) + \frac{1}{\delta}IU_{i,2} - \underbrace{\frac{1}{\alpha}S_{i,2}}_{(14)} + \underbrace{\sum_j \frac{1}{\kappa_{i,j}}(S_{j,2} - S_{i,2})}_{(15)}$$

$$\underbrace{\sum_j \frac{1}{\varpi_{i,j}}S_{j,3}}_{(16)} + \underbrace{\frac{1}{\zeta_{i,6}}S_{6,3}}_{(17)} - \mu S_{i,2}$$

$$\frac{dBT_{i,2}}{dt} = -\frac{1}{\sigma_2}BT_{i,2} - \frac{1}{r+\tau}BT_{i,2} - \frac{1}{\alpha}BT_{i,2} + \sum_j \frac{1}{\kappa_{i,j}}(BT_{j,2} - BT_{i,2}) + \sum_j \frac{1}{\varpi_{i,j}}BT_{j,3} + \frac{1}{\zeta_{i,6}}BT_{j,3} - \mu BT_{i,2}$$

$$\frac{dIT_{i,2}}{dt} = \frac{1}{\sigma_2}BT_{i,2} - \frac{1}{r+\tau}IT_{i,2} - \frac{1}{\alpha}IT_{i,2} + \sum_j \frac{1}{\kappa_{i,j}}(IT_{j,2} - IT_{i,2}) + \sum_j \frac{1}{\varpi_{i,j}}IT_{j,3} + \frac{1}{\zeta_{i,6}}IT_{j,3} - \mu IT_{i,2}$$

$$\frac{dBU_{i,2}}{dt} = -\frac{1}{\sigma_2}BU_{i,2} - \frac{1}{\alpha}BU_{i,2} + \sum_j \frac{1}{\kappa_{i,j}}(BU_{j,2} - BU_{i,2}) + \sum_j \frac{1}{\varpi_{i,j}}BU_{j,3} + \frac{1}{\zeta_{i,6}}BU_{j,3} - \mu BU_{i,2}$$

$$\frac{dIU_{i,2}}{dt} = \frac{1}{\sigma_2}BU_{i,2} - \frac{1}{\delta}IU_{i,2} - \frac{1}{\alpha}IU_{i,2} + \sum_j \frac{1}{\kappa_{i,j}}(IU_{j,2} - IU_{i,2}) + \sum_j \frac{1}{\varpi_{i,j}}IU_{j,3} + \frac{1}{\zeta_{i,6}}IU_{j,3} - \mu IU_{i,2}$$

(13) New infections arising from sub-patch 2 due to local transmission and not infections contracted while travelling

(14) Assimilation of population in sub-patch 2 (locals having returned from foreign travel) back into sub-patch 1 from whence they originated.

(15) Movement between local patches (1-5) out of and into the compartment

(16) Movement of patch 6 population from foreign patch j, sub-patch 3 back into patch 6 but in sub-patch 2, when i=6 and j = 1-5; =0 for all other values of i as this rate is particular to movement of Maputo population (patch 6)

(17) Movement of patch i population from foreign patch 6 sub-patch 3 back into patch i, sub-patch 2, when i=1-5; =0 for j=6 as this rate is particular to movement of the Mpumalanga population to and from Maputo (patches 1-5)

Sub-patch 3 (Foreign population): For each patch i moving to patch j $(i, j\ \epsilon \{1, 2, .., 6\}, j \neq i)$:

$$\frac{dS_{i,3}}{dt} = -\underbrace{\lambda_i[t-\sigma_1]seas_i[t]S_{i,3}}_{(18)} + \frac{1}{r+\tau}(BT_{i,3} + IT_{i,3}) + \frac{1}{\delta}IU_{i,3} + \underbrace{\sum_j \frac{1}{\kappa_{i,j}}(S_{j,3} - S_{i,3})}_{(19)}$$

$$+ \underbrace{\frac{1}{\varpi_{i,j}}(S_{j,1} - S_{i,3})}_{(20)} + \underbrace{\sum_j \frac{1}{\zeta_{i,j}}(S_{j,1} - S_{i,3})}_{(21)} - \mu S_{i,3}$$

$$\frac{dBT_{i,3}}{dt} = pf_y \lambda_i[t-\sigma_1] seas_i[t] S_{i,3} - \frac{1}{\sigma_2} BT_{i,3} - \frac{1}{r+\tau} BT_{i,3} + \sum_j \frac{1}{\kappa_{i,j}}(BT_{j,3} - BT_{i,3})$$

$$+ \frac{1}{\varpi_{i,j}}(BT_{j,1} - BT_{i,3}) + \sum_j \frac{1}{\zeta_{i,j}}(BT_{j,1} - BT_{i,3}) - \mu BT_{i,3}$$

$$\frac{dIT_{i,3}}{dt} = \frac{1}{\sigma_2} BT_{i,3} - \frac{1}{r+\tau} IT_{i,3} + \sum_j \frac{1}{\kappa_{i,j}}(IT_{j,3} - IT_{i,3}) + \frac{1}{\varpi_{i,j}}(IT_{j,1} - IT_{i,3}) + \sum_j \frac{1}{\zeta_{i,j}}(IT_{j,1} - IT_{i,3}) - \mu IT_{i,3}$$

$$\frac{dBU_{i,3}}{dt} = (1 - pf_y) \lambda_i[t-\sigma_1] seas_i[t] S_{i,3} - \frac{1}{\sigma_2} BU_{i,3} + \sum_j \frac{1}{\kappa_{i,j}}(BU_{j,3} - BU_{i,3}) + \frac{1}{\varpi_{i,j}}(BU_{j,1} - BU_{i,3})$$

$$+ \sum_j \frac{1}{\zeta_{i,j}}(BU_{j,1} - BU_{i,3}) - \mu BU_{i,3}$$

$$\frac{dIU_{i,3}}{dt} = \frac{1}{\sigma_2} BU_{i,3} - \frac{1}{\delta} IU_{i,3} + \sum_j \frac{1}{\kappa_{i,j}}(IU_{j,3} - IU_{i,3}) + \frac{1}{\varpi_{i,j}}(IU_{j,1} - IU_{i,3}) + \sum_j \frac{1}{\zeta_{i,j}}(IU_{j,1} - IU_{i,3}) - \mu IU_{i,3}$$

(18) New infections arising from sub-patch 3 due to local transmission and not infections contracted from patch of origin

(19) Movement between local patches (1-5) out of and into the compartment

(20) Movement of patch 6 population from patch 6, sub-patch 1 into patch i, sub-patch 3, when i=1-5 and j = 6 and movement from patch i, sub-patch 3 back into to patch 6, sub-patch 2. This rate =0 for all other values of j as it is particular to movement of Maputo population (patch 6)

(21) Movement of patch j population from patch j, sub-patch 1, into patch 6 sub-patch 3 when i=6 and j=1-5 and movement from patch 6, sub-patch 3 back into patch j, sub-patch 2 ; This rate=0 for j=6 as it is particular to movement of the Mpumalanga population (patches 1-5) to and from Maputo

Table 1: Model 2: Compartment Descriptions

Compartment	Description
$S_{i,k}$	Susceptible Population in patch i and sub-patch k
$BT_{i,k}$	Population with **Blood** Stage Infections in patch i and sub-patch k that are **treated**
$IT_{i,k}$	Population with **Infectious** Stage Infections in patch i and sub-patch k that are **treated**
$BU_{i,k}$	Population with **Blood** Stage Infections in patch i and sub-patch k that are **not treated**
$IU_{i,k}$	Population with **Infectious** Stage infections in patch i and sub-patch k that are **not treated**
$Smda_{i,k}$	Susceptible Population in patch i and sub-patch k receiving MDA
$Bmda_{i,k}$	Population with **Blood** stage infections in patch i and sub-patch k receiving MDA
$Imda_{i,k}$	Population with **Infectious** stage infections in patch i and sub-patch k receiving MDA
$Sfsat_{i,k}$	Susceptible Population in patch i and sub-patch k receiving FSAT
$Bfsat_{i,k}$	Population with **Blood** stage infections in patch i and sub-patch k receiving FSAT
$Ifsat_{i,k}$	Population with **Infectious** stage infections in patch i and sub-patch k receiving FSAT
$BT(mda)_{i,k}$	Population having already received MDA, with **Blood** Stage Infections in patch i and sub-patch k that are **treated**
$IT(mda)_{i,k}$	Population having already received MDA, with **Infectious** Stage Infections in patch i and sub-patch k that are **treated**
$BU(mda)_{i,k}$	Population having already received MDA, with **Blood** Stage Infections in patch i and sub-patch k that are **not treated**
$IU(mda)_{i,k}$	Population having already received MDA, with **Infectious** Stage infections in patch i and sub-patch k that are **not treated**

Table 2: Values, descriptions and sources of the parameters driving the base metapopulation model of transmission. ($i = \{TC; MB; UJ; NK; BB; MP\}$) Values in parentheses are the assumed ranges for the parameter sensitivity analysis.

Parameter	Description	Value	Source
	**Base Model Parameters**		
N	Population size for the six patches	2.5×10^6	[13, 14]
μ	Mortality/birth Rate	$\frac{105}{10000}$	[15]
δ	Natural recovery period	26 weeks (24, 28)	[16–18]
σ_1	Period between liver stage and blood-stage	7 days (5-10)	[19–21]
σ_2	Period between blood-stage and onset of gametocytemia	2 weeks (1.8, 2.2)	[16, 22]
r	AL elimination half-life	6 days (4, 8)	[23]
τ	Time to seek treatment	1/2 week	Expert opinion
p	Proportion of local infected population receiving treatment	0.95	[24, 25]
pf_{yr}	Proportion of foreign infected population that receive treatment in a local patch	$pf_1 = 0.5655(0.5652, 0.5658)$ (pre April 2005) $pf_2 = 0.5500$ $(0.5494, 0.5506)$ (post April 2005)	Estimated from model fitting process
$seas_i$	Seasonal forcing function	Derived from data	[26]

β_i	Annual number of mosquito bites per person x proportion of bites testing positive for sporozoites for patch i	$\beta_{TC} = 0.334\ (0.244, 0.425)$ $\beta_{MB} = 2.178\ (2.056, 2.300)$ $\beta_{UJ} = 0.805\ (0.700, 0.910)$ $\beta_{NK} = 1.330\ (1.310, 1.350)$ $\beta_{BB} = 8.304\ (7.903, 8.705)$ $\beta_{MP} = 94.999\ (93.327, 96.671)$	Estimated from model fitting process
$\frac{1}{\alpha}$	Rate of movement between sub-patch 2 and sub-patch 1	2 weeks^{-1}(1.75, 2.25)	Expert opinion
$\frac{1}{k}$	Rate of movement between 5 Mpumalanga municipalities	1/48.603 (1/51.328, 1/45.787) weeks^{-1}	Estimated from model fitting process
$\frac{1}{v_y}$	Maputo residents: Rate of movement between Maputo and 5 Mpumalanga municipalities	$\frac{1}{v_1} = 1/1258.828$ weeks^{-1} $(1/1261.249, 1/1256.407)$ (pre-April 2005) $\frac{1}{v_2} = 1/319.042$ weeks^{-1} $(1/322.796, 1/315.333)$ (post April 2005)	Estimated from model fitting process
$\frac{1}{z}$	Mpumalanga residents: Rate of movement between 5 Mpumalanga municipalities and Maputo	$\frac{1}{z} = 1/765.19$ weeks^{-1}	Estimated from model fitting process
$fwgt$	Foreign movement weight intensity	8.385 (8.232, 8.537)	Estimated from model fitting process
$lwgt$	Local movement weight intensity	2.613 (2.607, 2.618)	Estimated from model fitting process
$vc[i,t]$	$vccov[i,t] \times vef$		
$vccov[i,t]$	Vector Control Coverage	0.22-0.90	Derived from data
vef	Effectiveness of vector control	0.900 (0.897, 0.903)	Estimated from model fitting process
	**Scale up of Vector Control**		
$add_i[t]$	Additional Vector Control	$vcaddon \times vcadd_i$	
$vcaddon$	Additional Vector Control Switch	Binary	
$vcadd_i$	Additional Vector Control Coverage in patch i	10%, 20%	
	**Mass Drug Administration**		
$mrate[t]^{-1}$	Rate of MDA Take-up	mdaon(-log(1-mcov)/mdur)	
$mdaon$	Mass Drug Administration Switch	Binary	
$mcov$	MDA coverage	80%	
$mdur$	Duration of MDA cycle	8 weeks	
$pro_{MDA}[t]$	Drug Protection period	8 weeks	
	**Focal Screen and Treat**		

$msprop[t]$	Proportion Screened and Treated through Border Control	mson × mscov
$fsaton$	Focal Screen and Treat Switch	Binary
$mscov$	FSAT coverage × effectiveness	70%
$pro_{FSAT}[t]$	Drug Protection period	8 weeks

Summary Equations: Metapopulation Transmission model with interventions

Mass Drug Administration, Focal Screen and Treat, Scale-up of Vector Control

The base metapopulation model is expanded to include the impact of interventions, in particular, mass drug administration, focal screen and treat and a scale up of vector control (Figure 1). Scaling up vector control is modelled as an additional decrease to β_i. As described in the paper, the FSAT campaign is focused at a border entry point, where both the local and foreign populations are subject to a screening and treating campaign. Hence three compartments are introduced into the model in sub-patches 2 and 3 of each patch to account for locals and foreign travel into Mpumalanga. Sfsat, Bfsat and Ifsat represent the Susceptible, Blood and Infectious stages for a population that has received treatment through an FSAT campaign. To include MDA, three compartments are introduced into the model in each sub-patch of each patch. Smda, Bmda and Imda represent the Susceptible, Blood and Infectious stages for a population that has received treatment through an MDA cycle. As MDA is subjected to a population regardless of disease status, all stages of infection must be accounted for. The period of chemoprophylaxis (the drug protection period) is often shorter than the duration of the MDA cycle, and given that individuals in the population can expect to receive MDA only once per cycle, it is necessary to allow for new infections during the MDA cycle for the population who has already received MDA, but are reinfected as the drug protection period has lapsed. BT(mda), IT(mda), BU(mda) and IU(mda) are included to account for these infections.

This leads to the following set of differential equations.

Sub-patch 1 (Local population): For each patch i moving to patch j $(i, j\ \epsilon \{1, 2, .., 6\}, j \neq i)$:

$$\frac{dS_{i,1}}{dt} = \mu N_i - \lambda_i[t-\sigma_1]seas_i[t]S_{i,1} + \frac{1}{r+\tau}(BT_{i,1} + IT_{i,1}) + \frac{1}{\delta}IU_{i,1} + \frac{1}{\alpha}S_{i,2} + \sum_j \frac{1}{\kappa_{i,j}}(S_{j,1} - S_{i,1})$$
$$- \frac{1}{\varpi_{i,j}}S_{i,1} - \frac{1}{\zeta_{i,6}}S_{i,1} - \underbrace{\frac{1}{mrate[t]}S_{i,1}}_{(22)} + \underbrace{\frac{1}{pro_{MDA}[t]}Smda_{i,1}}_{(23)} - \mu S_{i,1}$$

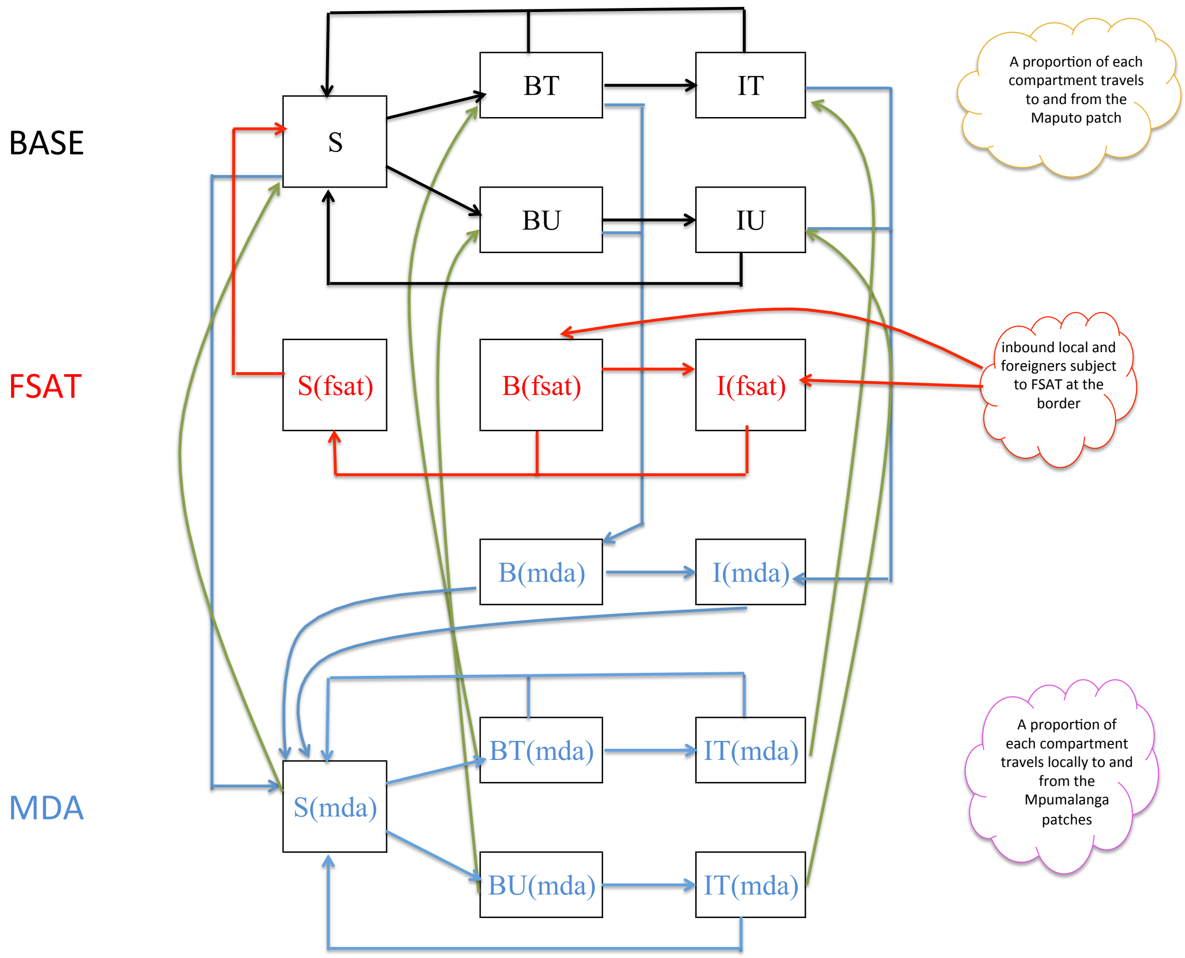

Figure 1: Transmission model with interventions for a single patch. Migration flows between patches are not shown. Note that the full model may be depicted as this single patch model, replicated 18 times, with migration flows between the 18 patches as described in the paper.

$$
\begin{aligned}
\frac{dBT_{i,1}}{dt} &= p\lambda_i[t-\sigma_1]seas_i[t](S_{i,1}+S_{i,2}) - \frac{1}{\sigma_2}BT_{i,1} - \frac{1}{r+\tau}BT_{i,1} + \frac{1}{\alpha}BT_{i,2} + \sum_j \frac{1}{\kappa_{i,j}}(BT_{j,1}-BT_{i,1}) \\
&- \frac{1}{\varpi_{i,j}}BT_{i,1} - \frac{1}{\zeta_{i,6}}BT_{i,1} - \frac{1}{mrate[t]}BT_{i,1} + \underbrace{\frac{1}{pro_{MDA}[t]}BT(mda)_{i,1}}_{(24)} - \mu BT_{i,1} \\
\frac{dIT_{i,1}}{dt} &= \frac{1}{\sigma_2}BT_{i,1} - \frac{1}{r+\tau}IT_{i,1} + \frac{1}{\alpha}IT_{i,2} + \sum_j \frac{1}{\kappa_{i,j}}(IT_{j,1}-IT_{i,1}) - \frac{1}{\varpi_{i,j}}IT_{i,1} - \frac{1}{\zeta_{i,6}}IT_{i,1} \\
&- \frac{1}{mrate[t]}IT_{i,1} + \frac{1}{pro_{MDA}[t]}IT(mda)_{i,1} - \mu IT_{i,1} \\
\frac{dBU_{i,1}}{dt} &= (1-p)\lambda_i[t-\sigma_1]seas_i[t](S_{i,1}+S_{i,2}) - \frac{1}{\sigma_2}BU_{i,1} + \frac{1}{\alpha}BU_{i,2} + \sum_j \frac{1}{\kappa_{i,j}}(BU_{j,1}-BU_{i,1}) \\
&- \frac{1}{\varpi_{i,j}}BU_{i,1} - \frac{1}{\zeta_{i,6}}BU_{i,1} - \frac{1}{mrate[t]}BU_{i,1} + \frac{1}{pro_{MDA}[t]}BU(mda)_{i,1} - \mu BU_{i,1} \\
\frac{dIU_{i,1}}{dt} &= \frac{1}{\sigma_2}BU_{i,1} - \frac{1}{\delta}IU_{i,1} + \frac{1}{\alpha}IU_{i,2} + \sum_j \frac{1}{\kappa_{i,j}}(IU_{j,1}-IU_{i,1}) - \frac{1}{\varpi_{i,j}}IU_{i,1} - \frac{1}{\zeta_{i,6}}IU_{i,1} \\
&- \frac{1}{mrate[t]}IU_{i,1} + \frac{1}{pro_{MDA}[t]}IU(mda)_{i,1} - \mu IU_{i,1} \\
\frac{dSmda_{i,1}}{dt} &= -\lambda_i[t-\sigma_1]seas_i[t]Smda_{i,1} + \frac{1}{mrate[t]}S_{i,1} + \underbrace{\frac{1}{r}(Bmda_{i,1}+Imda_{i,1})}_{(25)} - \frac{1}{pro_{MDA}[t]}Smda_{i,1} \\
&- \mu Smda_{i,1} \\
\frac{dBmda_{i,1}}{dt} &= \frac{1}{mrate[t]}(BT_{i,1}+BU_{i,1}) - \frac{1}{\sigma_2}Bmda_{i,1} - \frac{1}{r}Bmda_{i,1} - \mu Bmda_{i,1} \\
\frac{dImda_{i,1}}{dt} &= \frac{1}{mrate[t]}(IT_{i,1}+IU_{i,1}) + \frac{1}{\sigma_2}Bmda_{i,1} - \frac{1}{r}Imda_{i,1} - \mu Imda_{i,1} \\
\frac{dSfsat_{i,1}}{dt} &= \underbrace{0}_{(26)} \\
\frac{dBfsat_{i,1}}{dt} &= \underbrace{0}_{(26)} \\
\frac{dIfsat_{i,1}}{dt} &= \underbrace{0}_{(26)} \\
\frac{dBT(mda)_{i,1}}{dt} &= p\lambda_i[t-\sigma_1]seas_i[t](Smda_{i,1}+Smda_{i,2}) - \frac{1}{\sigma_2}BT(mda)_{i,1} - \frac{1}{r+\tau}BT(mda)_{i,1} + \frac{1}{\alpha}BT(mda)_{i,2} + \\
&\sum_j \frac{1}{\kappa_{i,j}}(BT(mda)_{j,1}-BT(mda)_{i,1}) - \frac{1}{\varpi_{i,j}}BT(mda)_{i,1} - \frac{1}{\zeta_{i,6}}BT(mda)_{i,1} - \frac{1}{pro_{MDA}[t]}BT(mda)_{i,1} \\
&- \mu BT(mda)_{i,1} \\
\frac{dIT(mda)_{i,1}}{dt} &= \frac{1}{\sigma_2}BT(mda)_{i,1} - \frac{1}{r+\tau}IT(mda)_{i,1} + \frac{1}{\alpha}IT(mda)_{i,2} + \sum_j \frac{1}{\kappa_{i,j}}(IT(mda)_{j,1}-IT(mda)_{i,1}) \\
&- \frac{1}{\varpi_{i,j}}IT(mda)_{i,1} - \frac{1}{\zeta_{i,6}}IT(mda)_{i,1} - \frac{1}{pro_{MDA}[t]}IT(mda)_{i,1} - \mu IT(mda)_{i,1}
\end{aligned}
$$

$$\frac{dBU(mda)_{i,1}}{dt} = (1-p)\lambda_i[t-\sigma_1]seas_i[t](Smda_{i,1}+Smda_{i,2}) - \frac{1}{\sigma_2}BU(mda)_{i,1} + \frac{1}{\alpha}BU(mda)_{i,2}$$
$$+\sum_j \frac{1}{\kappa_{i,j}}(BU(mda)_{j,1} - BU(mda)_{i,1}) - \frac{1}{\varpi_{i,j}}BU(mda)_{i,1} - \frac{1}{\zeta_{i,6}}BU(mda)_{i,1}$$
$$-\frac{1}{pro_{MDA}[t]}BU(mda)_{i,1} - \mu BU(mda)_{i,1}$$
$$\frac{dIU(mda)_{i,1}}{dt} = \frac{1}{\sigma_2}BU(mda)_{i,1} - \frac{1}{\delta}IU(mda)_{i,1} + \frac{1}{\alpha}IU(mda)_{i,2} + \sum_j \frac{1}{\kappa_{i,j}}(IU(mda)_{j,1} - IU(mda)_{i,1})$$
$$-\frac{1}{\varpi_{i,j}}IU(mda)_{i,1} - \frac{1}{\zeta_{i,6}}IU(mda)_{i,1} - \frac{1}{pro_{MDA}[t]}IU(mda)_{i,1} - \mu IU(mda)_{i,1}$$

(22) Take-up of MDA at a rate dependent on duration of the MDA cycle and the target coverage

(23) Assimilation back into the Susceptible population after MDA cycle is complete

(24) Recovery of infections treated during MDA

(25) Assimilation back into the Blood stage treated population after MDA cycle is complete

(26) As FSAT is modelled to be conducted at the Mpumalanga-Maputo border, only locals returning from Maputo (movement from sub-patch 3 to sub-patch 2) or foreigners entering Mpumalanga (movement from sub-patch 1 to sub-patch 3) are affected. Thus residents of sub-patch 1 are not affected by FSAT.

Sub-patch 2 (Local population returning from foreign travel): For each patch i moving to patch j $(i, j\ \epsilon\{1, 2, .., 6\}, j \neq i)$

$$\frac{dS_{i,2}}{dt} = -\lambda_i[t-\sigma_1]seas_i[t]S_{i,2} + \frac{1}{r+\tau}(BT_{i,2}+IT_{i,2}) + \frac{1}{\delta}IU_{i,2} - \frac{1}{\alpha}S_{i,2} + \sum_j \frac{1}{\kappa_{i,j}}(S_{j,2} - S_{i,2})$$
$$+\sum_j \frac{1}{\varpi_{i,j}}S_{j,3} + \frac{1}{\zeta_{i,6}}S_{j,3} - \frac{1}{mrate[t]}S_{i,2} + \frac{1}{pro_{MDA}[t]}Smda_{i,2} + \underbrace{\frac{1}{pro_{FSAT}[t]}Sfsat_{i,2}}_{(27)} - \mu S_{i,2}$$
$$\frac{dBT_{i,2}}{dt} = -\frac{1}{\sigma_2}BT_{i,2} - \frac{1}{r+\tau}BT_{i,2} - \frac{1}{\alpha}BT_{i,2} + \sum_j \frac{1}{\kappa_{i,j}}(BT_{j,2} - BT_{i,2}) + \underbrace{(1 - fsaton * mscov[t]) * \frac{1}{\zeta_{i,6}}BT_{j,3}}_{(28)}$$
$$+\underbrace{\sum_j \frac{1}{\varpi_{i,j}}BT_{j,3}}_{(29)} - \frac{1}{mrate[t]}BT_{i,2} + \frac{1}{pro_{MDA}[t]}BT(mda)_{i,2} - \mu BT_{i,2}$$
$$\frac{dIT_{i,2}}{dt} = \frac{1}{\sigma_2}BT_{i,2} - \frac{1}{r+\tau}IT_{i,2} - \frac{1}{\alpha}IT_{i,2} + \sum_j \frac{1}{\kappa_{i,j}}(IT_{j,2} - IT_{i,2}) + (1 - fsaton * mscov[t]) * \frac{1}{\zeta_{i,6}}IT_{j,3}$$
$$\sum_j \frac{1}{\varpi_{i,j}}IT_{j,3} - \frac{1}{mrate[t]}IT_{i,2} + \frac{1}{pro_{MDA}[t]}IT(mda)_{i,2} - \mu IT_{i,2}$$
$$\frac{dBU_{i,2}}{dt} = -\frac{1}{\sigma_2}BU_{i,2} - \frac{1}{\alpha}BU_{i,2} + \sum_j \frac{1}{\kappa_{i,j}}(BU_{j,2} - BU_{i,2}) + (1 - fsaton * mscov[t]) * \frac{1}{\zeta_{i,6}}BU_{j,3}$$
$$\sum_j \frac{1}{\varpi_{i,j}}BU_{j,3} - \frac{1}{mrate[t]}BU_{i,2} + \frac{1}{pro_{MDA}[t]}BU(mda)_{i,2} - \mu BU_{i,2}$$

$$
\begin{aligned}
\frac{dIU_{i,2}}{dt} &= \frac{1}{\sigma_2}BU_{i,2} - \frac{1}{\delta}IU_{i,2} - \frac{1}{\alpha}IU_{i,2} + \sum_j \frac{1}{\kappa_{i,j}}(IU_{j,2} - IU_{i,2}) + (1 - fsaton * mscov[t]) * \frac{1}{\zeta_{i,6}}IU_{j,3} \\
&\sum_j \frac{1}{\varpi_{i,j}}IU_{j,3} - \frac{1}{mrate[t]}IU_{i,2} + \frac{1}{pro_{MDA}[t]}IU(mda)_{i,2} - \mu IU_{i,2} \\
\frac{dSmda_{i,2}}{dt} &= -\lambda_i[t-\sigma_1]seas_i[t]Smda_{i,2} + \frac{1}{mrate[t]}S_{i,2} + \frac{1}{r}(Bmda_{i,2} + Imda_{i,2}) - \frac{1}{pro_{MDA}[t]}Smda_{i,2} \\
&- \mu Smda_{i,2} \\
\frac{dBmda_{i,2}}{dt} &= \frac{1}{mrate[t]}(BT_{i,2} + BU_{i,2}) - \frac{1}{\sigma_2}Bmda_{i,2} - \frac{1}{r}Bmda_{i,2} - \mu Bmda_{i,2} \\
\frac{dImda_{i,2}}{dt} &= \frac{1}{mrate[t]}(IT_{i,2} + IU_{i,2}) + \frac{1}{\sigma_2}Bmda_{i,2} - \frac{1}{r}Imda_{i,2} - \mu Imda_{i,2} \\
\frac{dSfsat_{i,2}}{dt} &= \underbrace{\frac{1}{r}(Bfsat_{i,2} + Ifsat_{i,2})}_{(30)} - \frac{1}{pro_{FSAT}[t]}Sfsat_{i,2} - \mu Sfsat_{i,2} \\
\frac{dBfsat_{i,2}}{dt} &= \underbrace{fsaton * mscov[t] * (\frac{1}{\zeta_{i,6}}BT_{j,3} + \frac{1}{\zeta_{i,6}}BU_{j,3})}_{(31)} - \frac{1}{r}Bfsat_{i,2} - \frac{1}{\sigma_2}Bfsat_{i,2} - \mu Bfsat_{i,2} \\
\frac{dIfsat_{i,2}}{dt} &= fsaton * mscov[t] * (\frac{1}{\zeta_{i,6}}IT_{j,3} + \frac{1}{\zeta_{i,6}}IU_{j,3}) + \frac{1}{\sigma_2}Bfsat_{i,2} - \frac{1}{r}Ifsat_{i,2} - \mu Ifsat_{i,2} \\
\frac{dBT(mda)_{i,2}}{dt} &= -\frac{1}{\sigma_2}BT(mda)_{i,2} - \frac{1}{r+\tau}BT(mda)_{i,2} - \frac{1}{\alpha}BT(mda)_{i,2} + \sum_j \frac{1}{\kappa_{i,j}}(BT(mda)_{j,2} - BT(mda)_{i,2}) \\
&+ \underbrace{(1 - fsaton * mscov[t]) * \frac{1}{\zeta_{i,6}}BT(mda)_{j,3}}_{(28)} + \underbrace{\sum_j \frac{1}{\varpi_{i,j}}BT(mda)_{j,3}}_{(29)} - \frac{1}{pro_{MDA}[t]}BT(mda)_{i,2} \\
&- \mu BT(mda)_{i,2} \\
\frac{dIT(mda)_{i,2}}{dt} &= \frac{1}{\sigma_2}BT(mda)_{i,2} - \frac{1}{r+\tau}IT(mda)_{i,2} - \frac{1}{\alpha}IT(mda)_{i,2} + \sum_j \frac{1}{\kappa_{i,j}}(IT(mda)_{j,2} - IT(mda)_{i,2}) \\
&+ (1 - fsaton * mscov[t]) * \frac{1}{\zeta_{i,6}}IT(mda)_{j,3} + \sum_j \frac{1}{\varpi_{i,j}}IT(mda)_{j,3} - \frac{1}{pro_{MDA}[t]}IT(mda)_{i,2} \\
&- \mu IT(mda)_{i,2} \\
\frac{dBU(mda)_{i,2}}{dt} &= -\frac{1}{\sigma_2}BU(mda)_{i,2} - \frac{1}{\alpha}BU(mda)_{i,2} + \sum_j \frac{1}{\kappa_{i,j}}(BU(mda)_{j,2} - BU(mda)_{i,2}) + \\
&(1 - fsaton * mscov[t]) * \frac{1}{\zeta_{i,6}}BU(mda)_{j,3} + \sum_j \frac{1}{\varpi_{i,j}}BU(mda)_{j,3} - \frac{1}{pro_{MDA}[t]}BU(mda)_{i,2} \\
&- \mu BU(mda)_{i,2} \\
\frac{dIU(mda)_{i,2}}{dt} &= \frac{1}{\sigma_2}BU(mda)_{i,2} - \frac{1}{\delta}IU(mda)_{i,2} - \frac{1}{\alpha}IU(mda)_{i,2} + \sum_j \frac{1}{\kappa_{i,j}}(IU(mda)_{j,2} - IU(mda)_{i,2}) \\
&+ (1 - fsaton * mscov[t]) * \frac{1}{\zeta_{i,6}}IU(mda)_{j,3} + \sum_j \frac{1}{\varpi_{i,j}}IU(mda)_{j,3} - \frac{1}{pro_{MDA}[t]}IU(mda)_{i,2} \\
&- \mu IU(mda)_{i,2}
\end{aligned}
$$

(27) Assimilation back into the Susceptible population after MDA cycle is complete

(28) Movement of patch i population **not subjected to FSAT** from foreign patch 6 sub-patch 3 back into patch i, sub-patch 2, when i=1-5; =0 for j=6 as this rate is particular to movement of the Mpumalanga population to and from Maputo (patches 1-5)

(29) Movement of patch 6 population from foreign patch j, sub-patch 3 back into patch 6 but in sub-patch 2, when i=6 and j = 1-5; =0 for all other values of i as this rate is particular to movement of Maputo population (patch 6)

(30) Recovery of infections treated during FSAT

(31) Take-up of FSAT (of locals entering Mpumalanga) at a rate dependent on duration of the FSAT cycle and the target coverage

Sub-patch 3 (Foreign population): For each patch i moving to patch j $(i, j \epsilon \{1, 2, .., 6\}, j \neq i)$:

$$
\begin{aligned}
\frac{dS_{i,3}}{dt} &= -\lambda_i[t-\sigma_1]seas_i[t]S_{i,3} + \frac{1}{r+\tau}(BT_{i,3} + IT_{i,3}) + \frac{1}{\delta}IU_{i,3} + \sum_j \frac{1}{\kappa_{i,j}}(S_{j,3} - S_{i,3}) \\
&+ \underbrace{\frac{1}{\varpi_{i,j}}(S_{j,1} - S_{i,3})}_{(32)} + \underbrace{\sum_j \frac{1}{\zeta_{i,j}}(S_{j,1} - S_{i,3})}_{(33)} - \frac{1}{mrate[t]}S_{i,3} + \frac{1}{pro_{MDA}[t]}Smda_{i,3} - \mu S_{i,3} \\
\frac{dBT_{i,3}}{dt} &= pf_y\lambda_i[t-\sigma_1]seas_i[t]S_{i,3} - \frac{1}{\sigma_2}BT_{i,3} - \frac{1}{r+\tau}BT_{i,3} + \sum_j \frac{1}{\kappa_{i,j}}(BT_{j,3} - BT_{i,3}) \\
&+ \underbrace{(1 - fsaton * mscov[t]) * \frac{1}{\varpi_{i,j}}BT_{j,1}}_{(34)} - \underbrace{\frac{1}{\varpi_{i,j}}BT_{i,3}}_{(35)} + \sum_j \frac{1}{\zeta_{i,j}}(BT_{j,1} - BT_{i,3}) - \frac{1}{mrate[t]}BT_{i,3} + \\
&\frac{1}{pro_{MDA}[t]}BT(mda)_{i,3} - \mu BT_{i,3} \\
\frac{dIT_{i,3}}{dt} &= \frac{1}{\sigma_2}BT_{i,3} - \frac{1}{r+\tau}IT_{i,3} + \sum_j \frac{1}{\kappa_{i,j}}(IT_{j,3} - IT_{i,3}) + (1 - fsaton * mscov[t]) * \frac{1}{\varpi_{i,j}}IT_{j,1} \\
&- \frac{1}{\varpi_{i,j}}IT_{i,3}) + \sum_j \frac{1}{\zeta_{i,j}}(IT_{j,1} - IT_{i,3}) - \frac{1}{mrate[t]}IT_{i,3} + \frac{1}{pro_{MDA}[t]}IT(mda)_{i,3} - \mu IT_{i,3} \\
\frac{dBU_{i,3}}{dt} &= (1 - pf_y)\lambda_i[t-\sigma_1]seas_i[t]S_{i,3} - \frac{1}{\sigma_2}BU_{i,3} + \sum_j \frac{1}{\kappa_{i,j}}(BU_{j,3} - BU_{i,3}) + \\
&(1 - fsaton * mscov[t]) * \frac{1}{\varpi_{i,j}}BU_{j,1} - \frac{1}{\varpi_{i,j}}BU_{i,3} + \sum_j \frac{1}{\zeta_{i,j}}(BU_{j,1} - BU_{i,3})) - \frac{1}{mrate[t]}BU_{i,3} + \\
&\frac{1}{pro_{MDA}[t]}BU(mda)_{i,3} - \mu BU_{i,3} \\
\frac{dIU_{i,3}}{dt} &= \frac{1}{\sigma_2}BU_{i,3} - \frac{1}{\delta}IU_{i,3} + \sum_j \frac{1}{\kappa_{i,j}}(IU_{j,3} - IU_{i,3}) + (1 - fsaton * mscov[t]) * \frac{1}{\varpi_{j,i}}IU_{j,1} \\
&- \frac{1}{\varpi_{j,i}}IU_{i,3}) + \sum_j \frac{1}{\zeta_{j,i}}(IU_{j,1} - IU_{i,3})) - \frac{1}{mrate[t]}IU_{i,3} + \frac{1}{pro_{MDA}[t]}IU(mda)_{i,3} - \mu IU_{i,3} \\
\frac{dSmda_{i,3}}{dt} &= -\lambda_i[t-\sigma_1]seas_i[t]S(mda)_{i,3} + \frac{1}{mrate[t]}S_{i,3} + \frac{1}{r}(Bmda_{i,3} + Imda_{i,3}) - \frac{1}{pro_{MDA}[t]}Smda_{i,3}
\end{aligned}
$$

$$
\begin{aligned}
&- \mu Smda_{i,3}\\
\frac{dBmda_{i,3}}{dt} &= \frac{1}{mrate[t]}(BT_{i,3} + BU_{i,3}) - \frac{1}{\sigma_2}Bmda_{i,3} - \frac{1}{r}Bmda_{i,3} - \mu Bmda_{i,3}\\
\frac{dImda_{i,3}}{dt} &= \frac{1}{mrate[t]}(IT_{i,3} + IU_{i,3}) + \frac{1}{\sigma_2}Bmda_{i,3} - \frac{1}{r}Imda_{i,3} - \mu Imda_{i,3}\\
\frac{dSfsat_{i,3}}{dt} &= \frac{1}{r}(Bfsat_{i,3} + Ifsat_{i,3}) - \frac{1}{pro_{FSAT}[t]}Sfsat_{i,3} - \mu Sfsat_{i,3}\\
\frac{dBfsat_{i,3}}{dt} &= \underbrace{fsaton * mscov[t] * (\frac{1}{\varpi_{i,j}}BT_{j,1} + \frac{1}{\varpi_{i,j}}BU_{j,1})}_{(36)} - \frac{1}{r}Bfsat_{i,3} - \frac{1}{\sigma_2}Bfsat_{i,3} - \mu Bfsat_{i,3}\\
\frac{dIfsat_{i,3}}{dt} &= fsaton * mscov[t] * (\frac{1}{\varpi_{i,j}}IT_{j,1} + \frac{1}{\varpi_{i,j}}IU_{j,1}) + \frac{1}{\sigma_2}Bfsat_{i,3} - \frac{1}{r}Ifsat_{i,3} - \mu Ifsat_{i,3}\\
\frac{dBT(mda)_{i,3}}{dt} &= pf_y\lambda_i[t-\sigma_1]seas_i[t]S(mda)_{i,3} - \frac{1}{\sigma_2}BT(mda)_{i,3} - \frac{1}{r+\tau}BT(mda)_{i,3} +\\
&\sum_j \frac{1}{\kappa_{i,j}}(BT(mda)_{j,3} - BT(mda)_{i,3}) + \underbrace{(1 - fsaton * mscov[t]) * \frac{1}{\varpi_{i,j}}BT(mda)_{j,1}}_{(34)}\\
&\underbrace{- \frac{1}{\varpi_{i,j}}BT(mda)_{i,3}}_{(35)} + \sum_j \frac{1}{\zeta_{i,j}}(BT(mda)_{j,1} - BT(mda)_{i,3}) - \frac{1}{pro_{MDA}[t]}BT(mda)_{i,3}\\
&- \mu BT(mda)_{i,3}\\
\frac{dIT(mda)_{i,3}}{dt} &= \frac{1}{\sigma_2}BT(mda)_{i,3} - \frac{1}{r+\tau}IT(mda)_{i,3} + \sum_j \frac{1}{\kappa_{i,j}}(IT(mda)_{j,3} - IT(mda)_{i,3}) +\\
&(1 - fsaton * mscov[t]) * \frac{1}{\varpi_{i,j}}IT(mda)_{j,1} - \frac{1}{\varpi_{i,j}}IT(mda)_{i,3}) + \sum_j \frac{1}{\zeta_{i,j}}(IT(mda)_{j,1} - IT_{i,3})\\
&- \frac{1}{pro_{MDA}[t]}IT(mda)_{i,3} - \mu IT(mda)_{i,3}\\
\frac{dBU(mda)_{i,3}}{dt} &= (1 - pf_y)\lambda_i[t-\sigma_1]seas_i[t]S(mda)_{i,3} - \frac{1}{\sigma_2}BU(mda)_{i,3} + \sum_j \frac{1}{\kappa_{i,j}}(BU(mda)_{j,3} - BU(mda)_{i,3})\\
&+ (1 - fsaton * mscov[t]) * \frac{1}{\varpi_{i,j}}BU(mda)_{j,1} - \frac{1}{\varpi_{i,j}}BU(mda)_{i,3}\\
&+ \sum_j \frac{1}{\zeta_{i,j}}(BU(mda)_{j,1} - BU(mda)_{i,3})) - \frac{1}{pro_{MDA}[t]}BU(mda)_{i,3} - \mu BU(mda)_{i,3}\\
\frac{dIU(mda)_{i,3}}{dt} &= \frac{1}{\sigma_2}BU(mda)_{i,3} - \frac{1}{\delta}IU(mda)_{i,3} + \sum_j \frac{1}{\kappa_{i,j}}(IU(mda)_{j,3} - IU(mda)_{i,3}) +\\
&(1 - fsaton * mscov[t]) * \frac{1}{\varpi_{j,i}}IU(mda)_{j,1} - \frac{1}{\varpi_{j,i}}IU(mda)_{i,3}) + \sum_j \frac{1}{\zeta_{j,i}}(IU(mda)_{j,1} - IU_{i,3}))\\
&- \frac{1}{pro_{MDA}[t]}IU(mda)_{i,3} - \mu IU(mda)_{i,3}
\end{aligned}
$$

(32) Movement of patch 6 population from patch 6, sub-patch 1 into patch i, sub-patch 3, when i=1-5 and j = 6 and movement from patch i, sub-patch 3 back into to patch 6, sub-patch 2. This rate =0 for all other values

of j as it is particular to movement of Maputo population (patch 6)

(33) Movement of patch j population from patch j, sub-patch 1, into patch 6 sub-patch 3 when i=6 and j=1-5 and movement from patch 6, sub-patch 3 back into patch j, sub-patch 2 ; This rate=0 for j=6 as it is particular to movement of the Mpumalanga population (patches 1-5) to and from Maputo

(34) Movement of patch 6 population **not subjected to FSAT** from patch 6, sub-patch 1 into patch i, sub-patch 3, when i=1-5 and j = 6. This rate =0 for all other values of j as it is particular to movement of Maputo population (patch 6)

(35) Movement from patch i, sub-patch 3 back into to patch 6, sub-patch 2 when i=1-5 and j = 6. This rate =0 for all other values of j as it is particular to movement of Maputo population (patch 6)

(36) Take-up of FSAT (of foreigners entering Mpumalanga) at a rate dependent on duration of the FSAT cycle and the target coverage

where the force of infection is given by

$$\lambda_i[t] = (1 - vc_i[t] * vef)\beta_i \times \frac{\sum_{k=1}^{3}(IT_{i,k}[t-4] + IU_{i,k} + Imda_{i,k}[t-4] + Ifsat_{i,k}[t-4])}{N_i}.$$

Vector Control

Indoor Residual spraying is the primary vector control intervention employed in Mpumalanga. The data on the number of structures sprayed in Mpumalanga is provided by the Malaria Elimination Programme and has already been presented in Ngomane and de Jager (2012) and is depicted in Figure 2 [34]. Given that IRS is not 100% effective, a parameter on the effectiveness of IRS vef has been estimated in the data-fitting process.

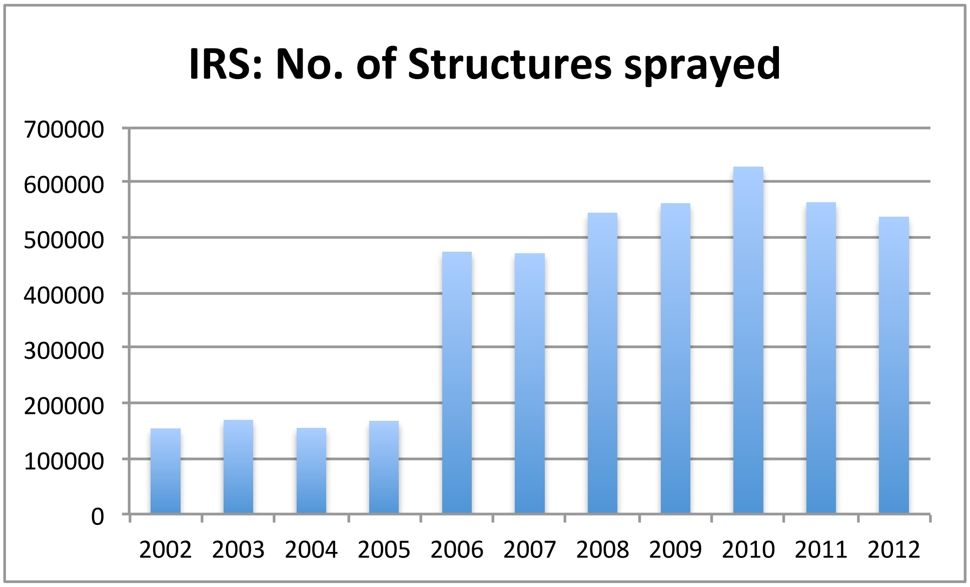

Figure 2: Number of structures sprayed in Mpumalanga between 2002 and 2012

Data Fitting Method

The model is fitted to weekly incidence data of treated cases from 2002 to 2008, and then validated with data from 2009 to 2012. The model is run from 1990 to reach a steady state before being fitted to data from 2002. IRS coverage and drug treatment are included in the model for the data fitting. The number of treated cases in each sub-patch k are fitted to the data using the maximum likelihood approach assuming an underlying Poisson distribution with canonical parameter λ as the average number of treated cases per week. The metapopulation non-linear differential equation model is expressed in terms of average rates of movement between compartments, thus λ is a function of the parameters to be estimated (listed in Table 2)

The Poisson probability of observing x counts when the average number of counts per week is λ given by

$$P(x|\lambda) = \frac{\lambda^x \exp^{-\lambda}}{x!}.$$

As the model is being fitted to time series data with N time bins, λ, the expected number of counts per bin is a function of time. Assuming the independence of data from different time bins, the likelihood reduces to

$$L(\lambda_i|x_i) = \prod_{i=1}^{N} \frac{\lambda_i^{x_i} \exp^{-\lambda_i}}{x_i!}$$

and the log likelihood becomes

$$ln(L(\lambda_i|x_i)) = \sum_{i=1}^{N} x_i ln(\lambda_i) - \lambda_i - ln(x_i!).$$

The model is fitted to 16 sets of data for each weekly time bin: treated cases for three sub-patches in five Mpumalanga municipalities and treated cases for Maputo. Under the assumption of independence, the log likelihood to be maximised is

$$ln(L(\lambda_{s,i}|x_{s,i})) \propto \sum_{i=1}^{N} \sum_{s=1}^{16} x_{s,i} ln(\lambda_{s,i}) - \lambda_{s,i}$$

The log-likelihood is negated and minimised using the hydroPSO function implementing a version of the Particle Swarm Optimisation algorithm in the R package hydroPSO v0.3-3 [29, 30]. Particle Swarm Optimisation is a global stochastic optimisation technique initially inspired by social behaviour of birds

and fish [31, 32]. It shares similarities with evolutionary optimisation techniques like Genetic Algorithms (GA) but explores the multi-dimensional solution space on the basis of individual and global best-known "particle positions" without evolution operators. Problems are optimised by moving particles (the population of candidate solutions) around the search-space based on the particles' position and velocity. Particle movements are a function of local best positions and other best particle positions in the search-space. Thus the particles "swarm" towards the best solutions in the search-space.

The parameters estimated through the model fitting process are presented in Table 2. The model with the estimated parameter values is run for a further 3 years to be further validated by comparison to data between 2009 and 2012. Model development, fitting and subsequent analysis was performed in R v3.02 [33]. The particle swarm optimization routine was performed using the R package hydroPSO v0.3-3 [29, 30]. Figure 3 shows the data fitting and validation for all 16 sets of data with 95% confidence intervals. The differential equation model predicts the *average* number of treated cases per time point, whereas the data is one observation in time. Running the model several times, sampling parameter estimates from their 95% confidence ranges and treating model flows as a random realisation of a Poisson distribution, the average prediction at each time point is computed and plotted (solid red and blue lines). The shaded envelope around the average prediction is a 95% pseudo confidence interval for an individual prediction (computed by averaging the lower and upper bounds over each of the 95% confidence intervals calculated for every model run) as opposed to an interval for the average prediction. The latter, by default, would be narrower. The model fits the data in sub-patches 1 and 2 well, capturing the level and timing of transmission. Sub-patch 3 data is over-estimated before 2006 and captured relatively well thereafter for all municipalities except Nkomazi, where the prediction of infections is over-estimated. Sub-patch 3 for Bushbuckridge appears to be over-estimated, but as the scale of the graph is small compared to Nkomazi, this is less of an "over"-estimation. Both the timing and level of malaria transmission in Maputo is captured by the model fitting process.

The model is made stochastic by treating each flow between compartments at time t as a random realisation of a Poisson process with rate λ as the deterministic flow value at that time and by simulating the parameter values from their 95% confidence intervals.

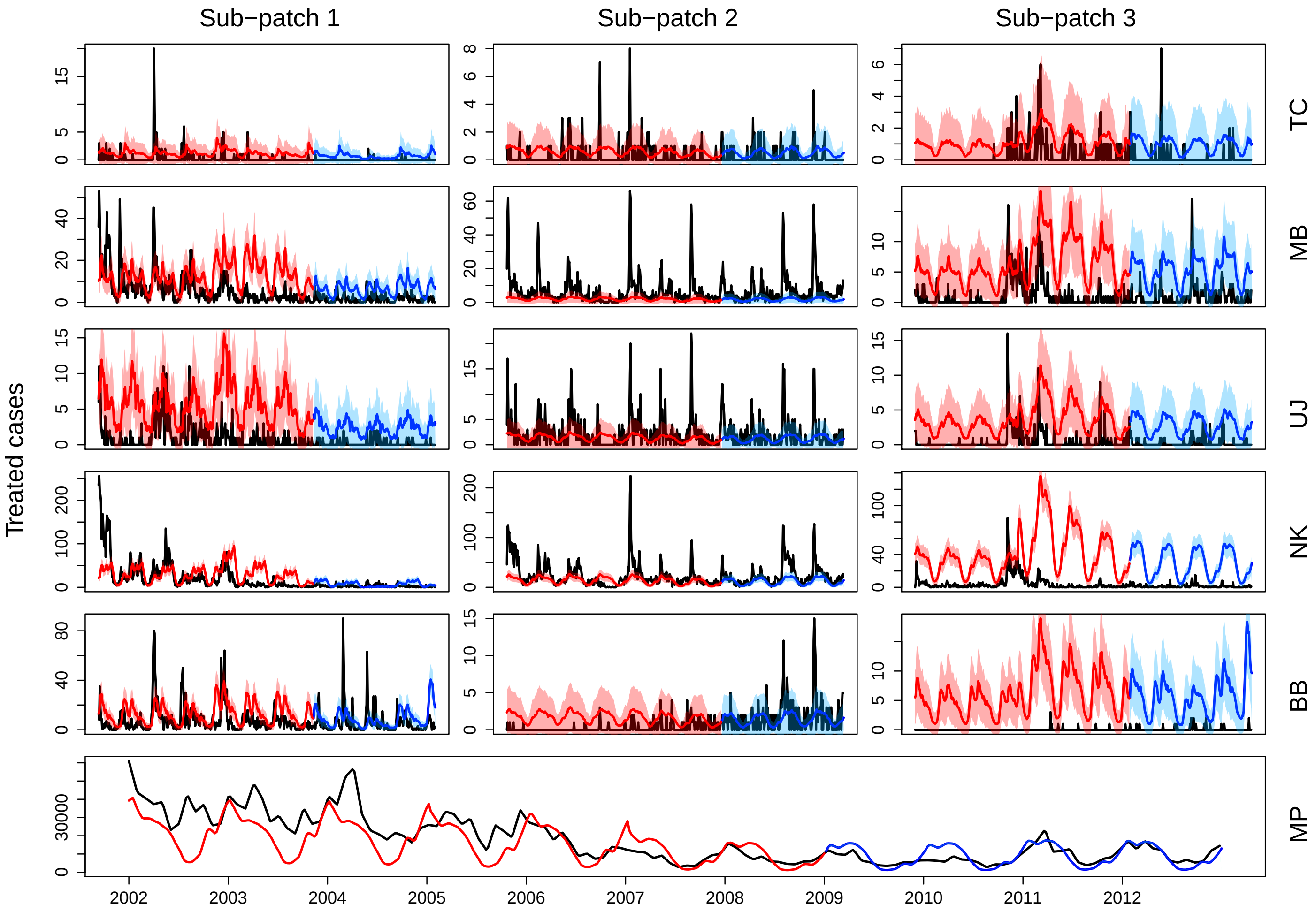

Figure 3: Modelling fit and validation with 95% uncertaintly range for individual predicitons

Migration rate sensitivity analysis

A test on the sensitivity of model predictions to changes in the migration rate was conducted. The model results as depicted in Figure 4 in the manuscript have been simulated at different levels of migration (-90%, -50%, -25%, base case, +25%, +50%, +100%). Figure 4 shows that the model predictions are stable for varying levels of migration for all interventions modelled.

Additional results

The graphs below depict the impact of the various interventions on infections in each of the five local municipalities.

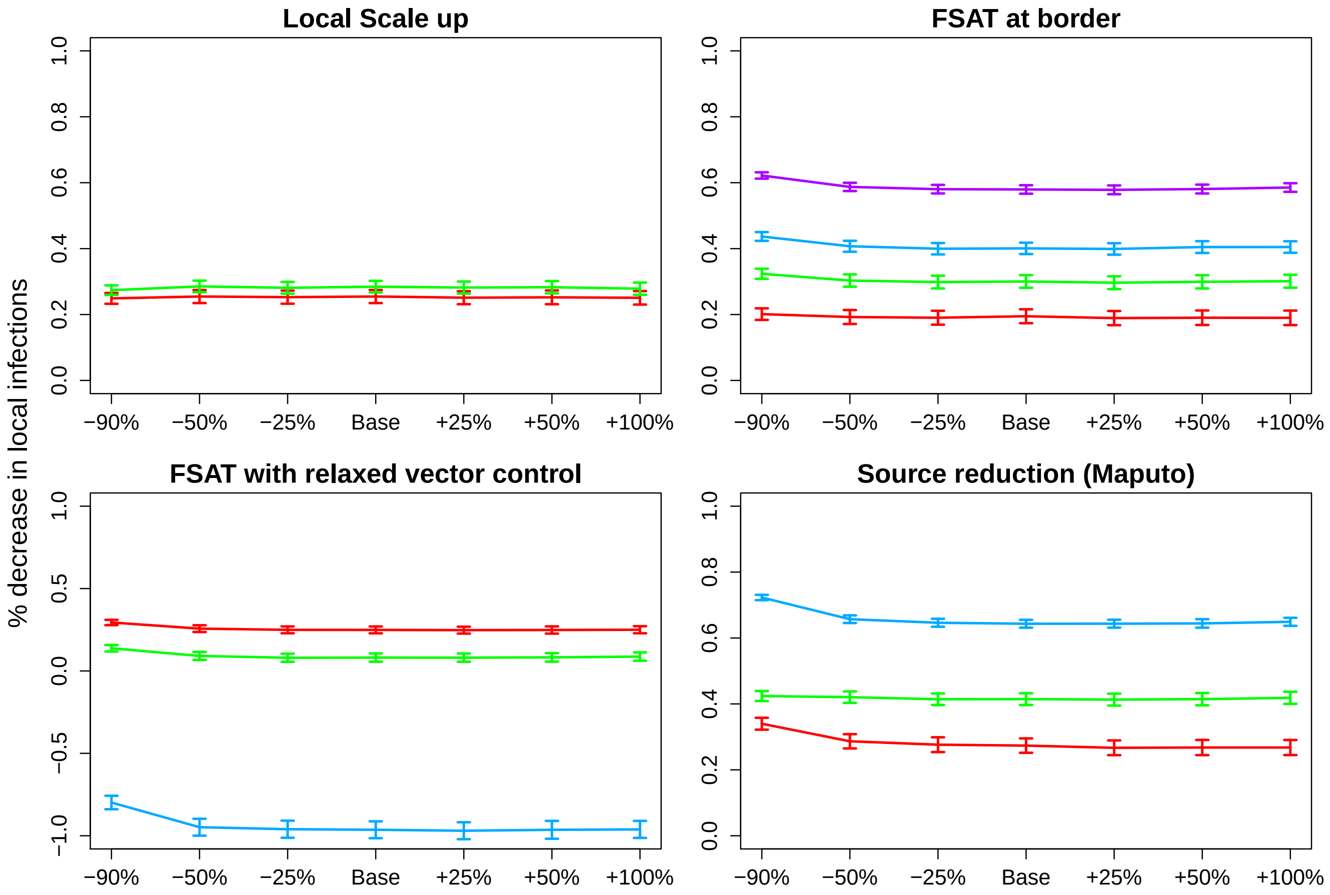

Figure 4: Predicted percentage change (increase or decrease) in point estimates of local infections due to the interventions between 2013 and 2018 for varying levels of the foreign migration rate. (1) Local Scale-up: Increase in local vector control so as to reduce the mosquito-human contact rate by a further 10% (red)& three consecutive two-monthly rounds of MDA in Mbombela, Nkomazi and Bushbuckridge Municipalities (green). (2) FSAT at the border: at 70% coverage for 26 weeks (red), 39 weeks (green), 52 weeks (blue) and 52 weeks at 100% coverage (purple). (3) Reducing Vector Control: FSAT at the border at 70% coverage administered all year round while simultaneously reducing vector control by 10% (red), 20% (green) and stopping vector control altogether (blue). (4) Source Reduction: 10% scale up of vector control in Maputo (red), three consecutive two-monthly rounds of MDA in Maputo (green) and eliminating malaria in Maputo (blue).

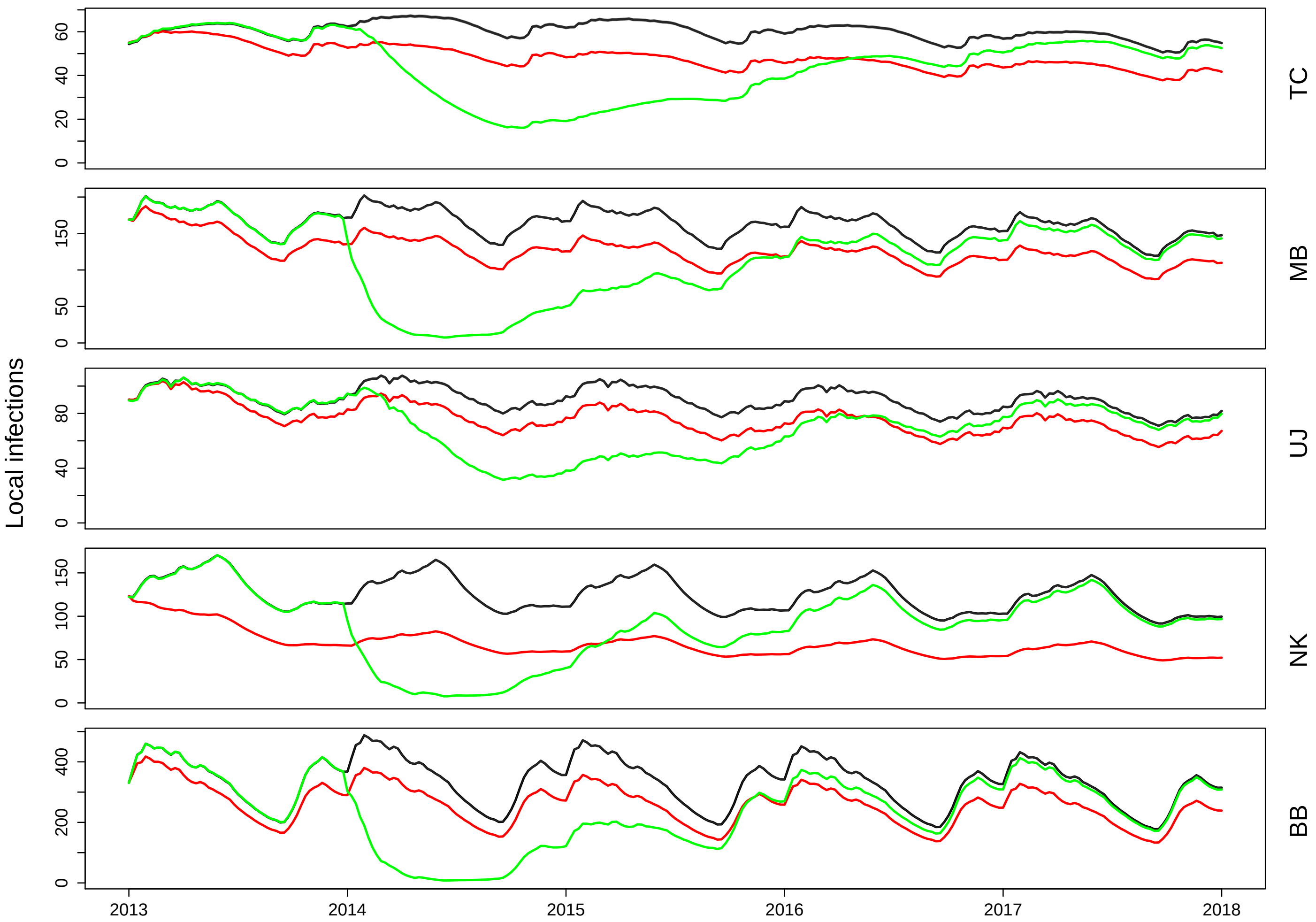

Figure 5: Predicted impact of interventions on local infections in the five local municipalities through time compared to the base case of no interventions (black). Local Scale-up: Increase in local vector control so as to reduce the mosquito-human contact rate by a further 10% (red)& three consecutive two-monthly rounds of MDA in Mbombela, Nkomazi and Bushbuckridge Municipalities (green).

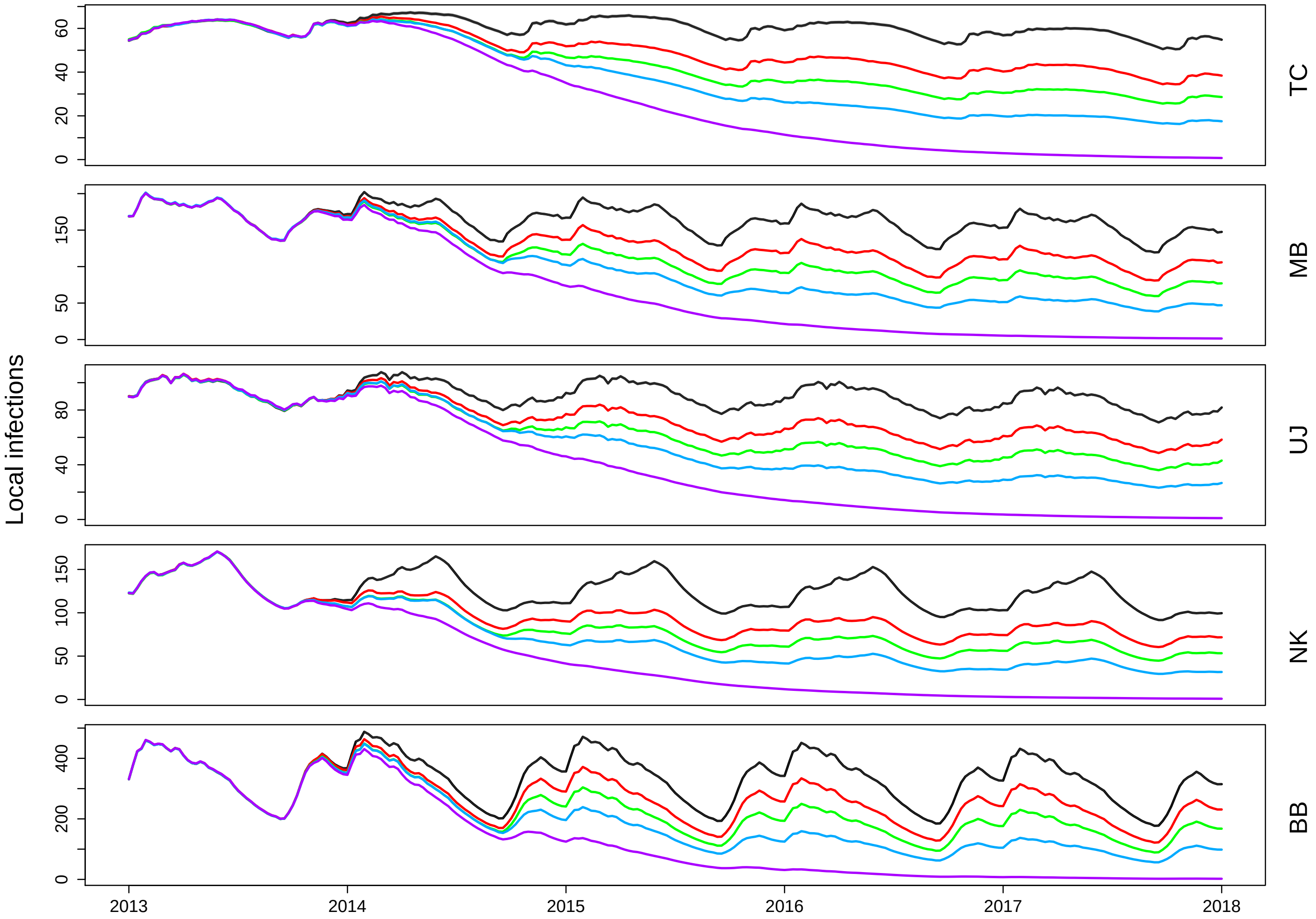

Figure 6: Predicted impact of interventions on local infections in the five local municipalities through time compared to the base case of no interventions (black). FSAT at the border: at 70% coverage for 26 weeks (red), 39 weeks (green), 52 weeks (blue) and 52 weeks at 100% coverage (purple).

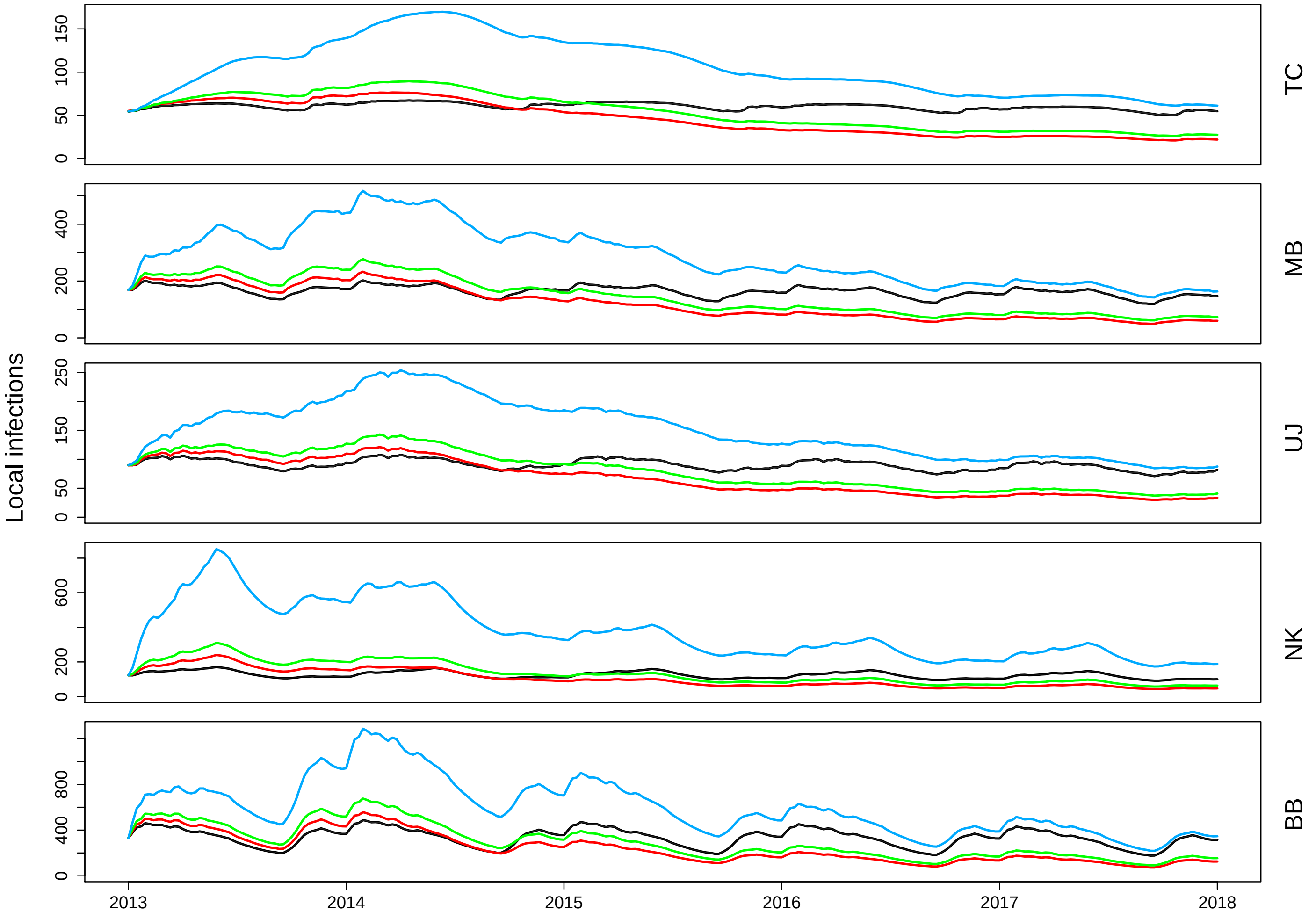

Figure 7: Predicted impact of interventions on local infections in the five local municipalities through time compared to the base case of no interventions (black). Reducing Vector Control: FSAT at the border at 70% coverage administered all year round while simultaneously reducing vector control by 10% (red), 20% (green) and stopping vector control altogether (blue).

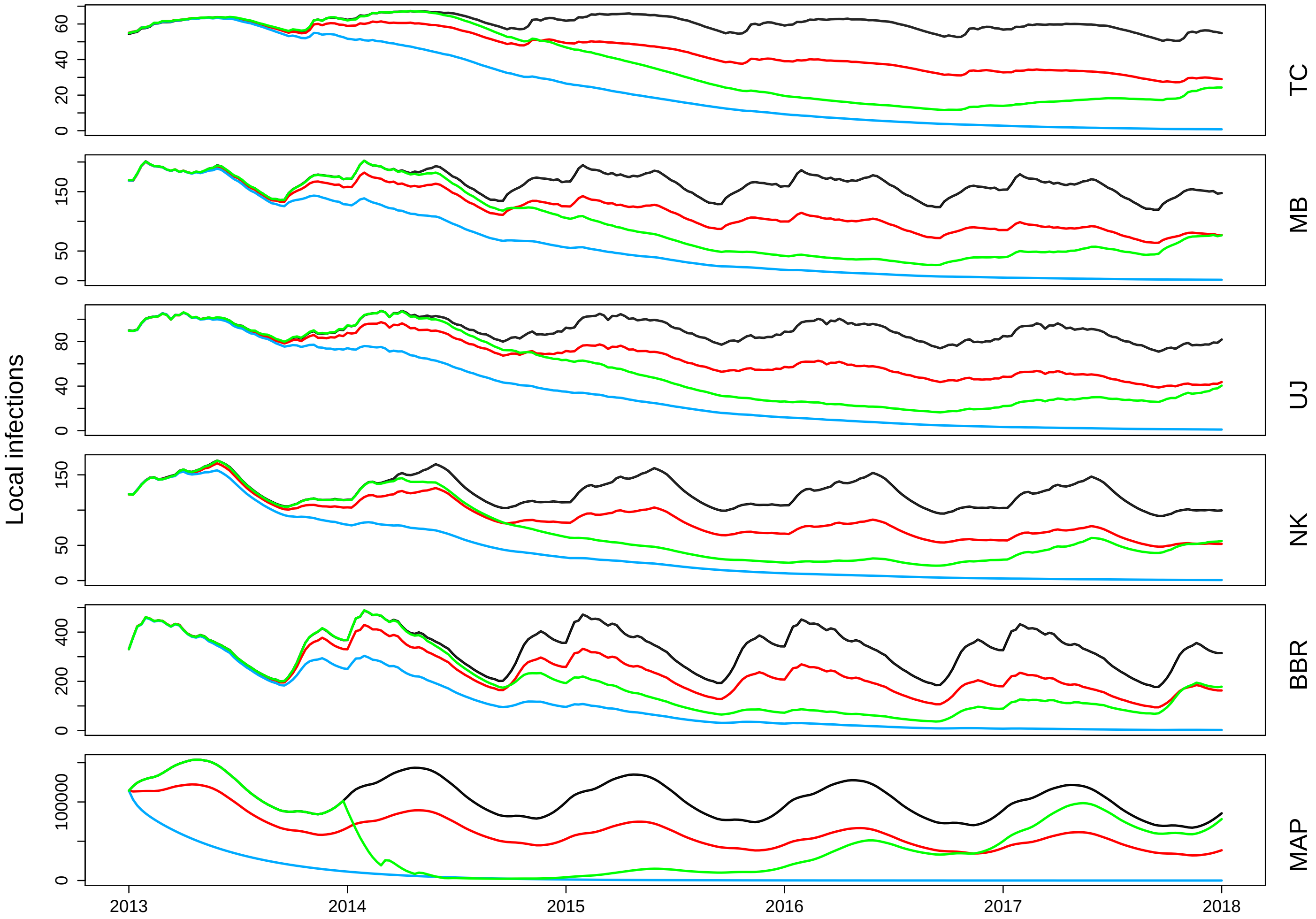

Figure 8: Predicted impact of interventions on local infections in the five local municipalities through time compared to the base case of no interventions (black). Source Reduction: 10% scale up of vector control in Maputo (red), three consecutive two-monthly rounds of MDA in Maputo (green) and eliminating malaria in Maputo (blue).

References

1. Ariey F, Duchemin JB, Robert V: **Metapopulation concepts applied to falciparum malaria and their impacts on the emergence and spread of chloroquine resistance.** *Infection, genetics and evolution : Journal of molecular epidemiology and evolutionary genetics in infectious diseases* 2003, **2**(3):185–92, [http://www.ncbi.nlm.nih.gov/pubmed/12797980].
2. Oluwagbemi OO, Fornadel CM, Adebiyi EF, Norris DE, Rasgon JL: **ANOSPEX: a stochastic, spatially explicit model for studying *Anopheles* metapopulation dynamics.** *PloS one* 2013, **8**(7), [http://www.plosone.org/article/info:doi/10.1371/journal.pone.0068040\#pone-0068040-g004].
3. Le Menach A, McKenzie FE, Flahault A, Smith DL: **The unexpected importance of mosquito oviposition behaviour for malaria: non-productive larval habitats can be sources for malaria transmission.** *Malaria Journal* 2005, **4**:23, [http://www.malariajournal.com/content/4/1/23].
4. Smith DL, Dushoff J, McKenzie FE: **The risk of a mosquito-borne infection in a heterogeneous environment.** *PLoS Biology* 2004, **2**(11), [http://dx.plos.org/10.1371/journal.pbio.0020368].
5. Arino J, Ducrot A, Zongo P: **A metapopulation model for malaria with transmission-blocking partial immunity in hosts.** *Journal of Mathematical Biology* 2012, **64**(3):423–48, [http://www.ncbi.nlm.nih.gov/pubmed/21442182].
6. Zorom M, Zongo P, Barbier B, Somé B: **Optimal control of a spatio-temporal model for Malaria: Synergy treatment and prevention**. *Journal of Applied Mathematics* 2012.
7. Auger P, Kouokam E, Sallet G, Tchuente M, Tsanou B: **The Ross-Macdonald model in a patchy environment.** *Mathematical Biosciences* 2008, **216**(2):123–31, [http://www.ncbi.nlm.nih.gov/pubmed/18805432].
8. Rodriguez DJ, Torres-Sorando L: **Models of infectious diseases in spatially heterogeneous environments.** *Bulletin of Mathematical Biology* 2001, **63**(3):547–71, [http://www.ncbi.nlm.nih.gov/pubmed/11374305].
9. Craig M, Snow R, le Sueur D: **A Climate-based Distribution Model of Malaria Transmission in Sub-Saharan Africa**. *Parasitology Today* 1999, **15**(3):105–111, [http://dx.doi.org/10.1016/S0169-4758(99)01396-4].
10. Montosi E, Manzoni S, Porporato A, Montanari A: **An ecohydrological model of malaria outbreaks**. *Hydrology and Earth System Sciences* 2012, **16**(8):2759–2769, [http://www.hydrol-earth-syst-sci.net/16/2759/2012/hess-16-2759-2012.html].
11. Coleman M, Coleman M, Mabuza AM, Kok G, Coetzee M, Durrheim DN: **Using the SaTScan method to detect local malaria clusters for guiding malaria control programmes.** *Malaria Journal* 2009, **8**:68, [http://www.malariajournal.com/content/8/1/68].
12. **Statistical release Mid-year population estimates**. Tech. rep., Statistics South Africa 2011, [http://www.statssa.gov.za/publications/P0302/P03022011.pdf].
13. Statistics South Africa (2012) Census 2011 Municipal report Mpumalanga Technical report, Statistics South Africa, Pretoria. http://www.statssa.gov.za/Census2011/Products/MP_Municipal_Report.pdf.
14. Zacarias, Orlando P, Andersson, Mikael (2010) Mapping malaria incidence distribution that accounts for environmental factors in Maputo Province-Mozambique Malaria Journal 9.
15. **Mortality and causes of death in South Africa, 2010: Findings from death notification**. Tech. rep., Statistics South Africa, Pretoria 2013, [http://www.statssa.gov.za/publications/p03093/p030932010.pdf].
16. Jeffery GM, Eyles DE: **Infectivity to mosquitoes of *Plasmodium falciparum* as related to gametocyte density and duration of infection.** *The American journal of Tropical Medicine and Hygiene* 1955, **4**(5):781–9, [http://www.ncbi.nlm.nih.gov/pubmed/13259002].
17. Miller MJ: **Observations on the natural history of malaria in the semi-resistant West African.** *Transactions of the Royal Society of Tropical Medicine and Hygiene* 1958, **52**:152–68, [http://www.ncbi.nlm.nih.gov/pubmed/13543904].
18. White LJ, Maude RJ, Pongtavornpinyo W, Saralamba S, Aguas R, Van Effelterre T, Day NPJ, White NJ: **The role of simple mathematical models in malaria elimination strategy design.** *Malaria Journal* 2009, **8**:212, [http://www.malariajournal.com/content/8/1/212].

19. Eyles DE, Young MD: **The duration of untreated or inadequately treated *Plasmodium falciparum* infections in the human host.** *Journal of National Malaria Society (U.S.)* 1951, **10**(4):327–36, [http://www.ncbi.nlm.nih.gov/pubmed/14908561].

20. Collins WE, Jeffery GM: **A retrospective examination of sporozoite- and trophozoite-induced infections with *Plasmodium falciparum*: development of parasitologic and clinical immunity during primary infection.** *The American Journal of Tropical Medicine and Hygiene* 1999, **61**(1 Suppl):4–19, [http://www.ncbi.nlm.nih.gov/pubmed/10432041].

21. Chitnis N, Hyman JM, Cushing JM: **Determining important parameters in the spread of malaria through the sensitivity analysis of a mathematical model.** *Bulletin of Mathematical Biology* 2008, **70**(5):1272–96, [http://www.ncbi.nlm.nih.gov/pubmed/18293044].

22. Thomson D: *A Research Into the Production, Life and Death of Crescents in Malignant Tertian Malaria, in Treated and Untreated Cases, by an Enumerative Method; The Leucocytes in Malarial Fever: A Method of Diagnosing Malaria Long After it is Apparently Cured*. University Press 1911.

23. Makanga M, Krudsood S: **The clinical efficacy of artemether/lumefantrine (Coartem).** *Malaria Journal* 2009, **8**(Suppl 1), [http://www.malariajournal.com/content/8/S1/S5].

24. Castillo-Riquelme M, McIntyre D, Barnes K: **Household burden of malaria in South Africa and Mozambique: is there a catastrophic impact?** *Tropical medicine and International health* 2008, **13**:108–22, [http://www.ncbi.nlm.nih.gov/pubmed/18291009].

25. Hlongwana KW, Zitha A, Mabuza AM, Maharaj R: **Knowledge and practices towards malaria amongst residents of Bushbuckridge, Mpumalanga, South Africa**. *African Journal of Primary Health Care & Family Medicine* 2011, **3**, [http://www.phcfm.org/index.php/phcfm/article/view/257].

26. Silal SP, Barnes KI, Kok G, Mabuza A, Little F: **Exploring the seasonality of reported treated malaria cases in Mpumalanga, South Africa.** *PloS one* 2013, **8**(10):e76640, [http://www.plosone.org/article/info\%3Adoi\%2F10.1371\%2Fjournal.pone.0076640].

27. of Home Affairs D: **South Africa and Mozambique sign a Visa Waiver Agreement**. Tech. rep., Department of Home Affairs 2005.

28. Cleveland RB: **STL: A seasonal-trend decomposition procedure based on LOESS**. *Journal of Official Statistics* 1990, **6**, [{http://cs.wellesley.edu/~cs315/Papers/stl\%20statistical\%20model.pdf}].

29. Zambrano-Bigiarini, M, Rojas, R: **A model-independent Particle Swarm Optimisation software for model calibration**. *Environmental Modelling and Software* 2013, **43**:5–25, [http://dx.doi.org/10.1016/j.envsoft.2013.01.004].

30. Zambrano-Bigiarini M, Rojas R: *hydroPSO: Particle Swarm Optimisation, with focus on Environmental Models* 2013, [http://www.rforge.net/hydroPSO,http://cran.r-project.org/web/packages/hydroPSO]. [R package version 0.3-3 — For new features, see the 'NEWS' file (on CRAN, rforge or the package source)].

31. Kennedy J, Eberhart R: **Particle swarm optimization**. In *Proceedings of ICNN'95 - International Conference on Neural Networks, Volume 4*, IEEE:1942–1948, [http://ieeexplore.ieee.org/articleDetails.jsp?arnumber=488968].

32. Eberhart R, Kennedy J: **A new optimizer using particle swarm theory**. In *MHS'95. Proceedings of the Sixth International Symposium on Micro Machine and Human Science*, IEEE 1995:39–43, [http://ieeexplore.ieee.org/articleDetails.jsp?arnumber=494215].

33. R Core Group: 2013, [www.r-project.org].

34. Ngomane L, de Jager C: **Changes in malaria morbidity and mortality in Mpumalanga Province, South Africa (2001- 2009): a retrospective study**. *Malaria Journal* 2012, **11**:19, [http://www.malariajournal.com/content/11/1/19].
